# Antibodies Targeting the Transferrin Receptor 1 (TfR1) as Direct Anti-cancer Agents

**DOI:** 10.3389/fimmu.2021.607692

**Published:** 2021-03-17

**Authors:** Pierre V. Candelaria, Lai Sum Leoh, Manuel L. Penichet, Tracy R. Daniels-Wells

**Affiliations:** ^1^Division of Surgical Oncology, Department of Surgery, David Geffen School of Medicine at the University of California, Los Angeles (UCLA), Los Angeles, CA, United States; ^2^Department of Microbiology, Immunology, and Molecular Genetics, David Geffen School of Medicine at UCLA, Los Angeles, CA, United States; ^3^Jonsson Comprehensive Cancer Center, UCLA, Los Angeles, CA, United States; ^4^The Molecular Biology Institute, UCLA, Los Angeles, CA, United States; ^5^UCLA AIDS Institute, UCLA, Los Angeles, CA, United States

**Keywords:** cancer, transferrin receptor, iron deprivation, CD71, antibody-mediated effector functions, immunotherapy

## Abstract

The transferrin receptor 1 (TfR1), also known as cluster of differentiation 71 (CD71), is a type II transmembrane glycoprotein that binds transferrin (Tf) and performs a critical role in cellular iron uptake through the interaction with iron-bound Tf. Iron is required for multiple cellular processes and is essential for DNA synthesis and, thus, cellular proliferation. Due to its central role in cancer cell pathology, malignant cells often overexpress TfR1 and this increased expression can be associated with poor prognosis in different types of cancer. The elevated levels of TfR1 expression on malignant cells, together with its extracellular accessibility, ability to internalize, and central role in cancer cell pathology make this receptor an attractive target for antibody-mediated therapy. The TfR1 can be targeted by antibodies for cancer therapy in two distinct ways: (1) indirectly through the use of antibodies conjugated to anti-cancer agents that are internalized by receptor-mediated endocytosis or (2) directly through the use of antibodies that disrupt the function of the receptor and/or induce Fc effector functions, such as antibody-dependent cell-mediated cytotoxicity (ADCC), antibody-dependent cell-mediated phagocytosis (ADCP), or complement-dependent cytotoxicity (CDC). Although TfR1 has been used extensively as a target for antibody-mediated cancer therapy over the years, interest continues to increase for both targeting the receptor for delivery purposes and for its use as direct anti-cancer agents. This review focuses on the developments in the use of antibodies targeting TfR1 as direct anti-tumor agents.

## Introduction

### The Relevance of Iron in Biology

Iron is a vital element in several biological processes including oxygen transportation, energy generation/mitochondrial function, as well as DNA synthesis and repair (1–3). Iron is a co-factor for the ribonucleotide reductase enzyme that mediates conversion of ribonucleotides into deoxyribonucleotides that are used in DNA synthesis (4). The iron containing subunit of this enzyme, R2, also has the ability to repair damaged DNA through a p53-mediated pathway (5). Within the context of a cancer cell, facilitating DNA synthesis would allow increased proliferation, while increased capacity to repair DNA would aid in repairing DNA damage from the increased mutational burden common among cancer cells (6). In addition, iron is used to make heme-containing proteins, such as hemoglobin that is important for progenitor cells of the erythroid lineage (7) and cytochromes that are important part in mitochondrial function (8). Central proteins in the regulation of iron metabolism are transferrin (Tf) and its receptors. As the main cellular importer of iron, transferrin receptor 1 (TfR1) function is essential to iron related processes and the uptake of Tf-bound iron through TfR1 is the main source of cellular iron import in general.

### The Transferrin Receptor 1 (TfR1)

#### TfR1 Structure

**Figure 1:**
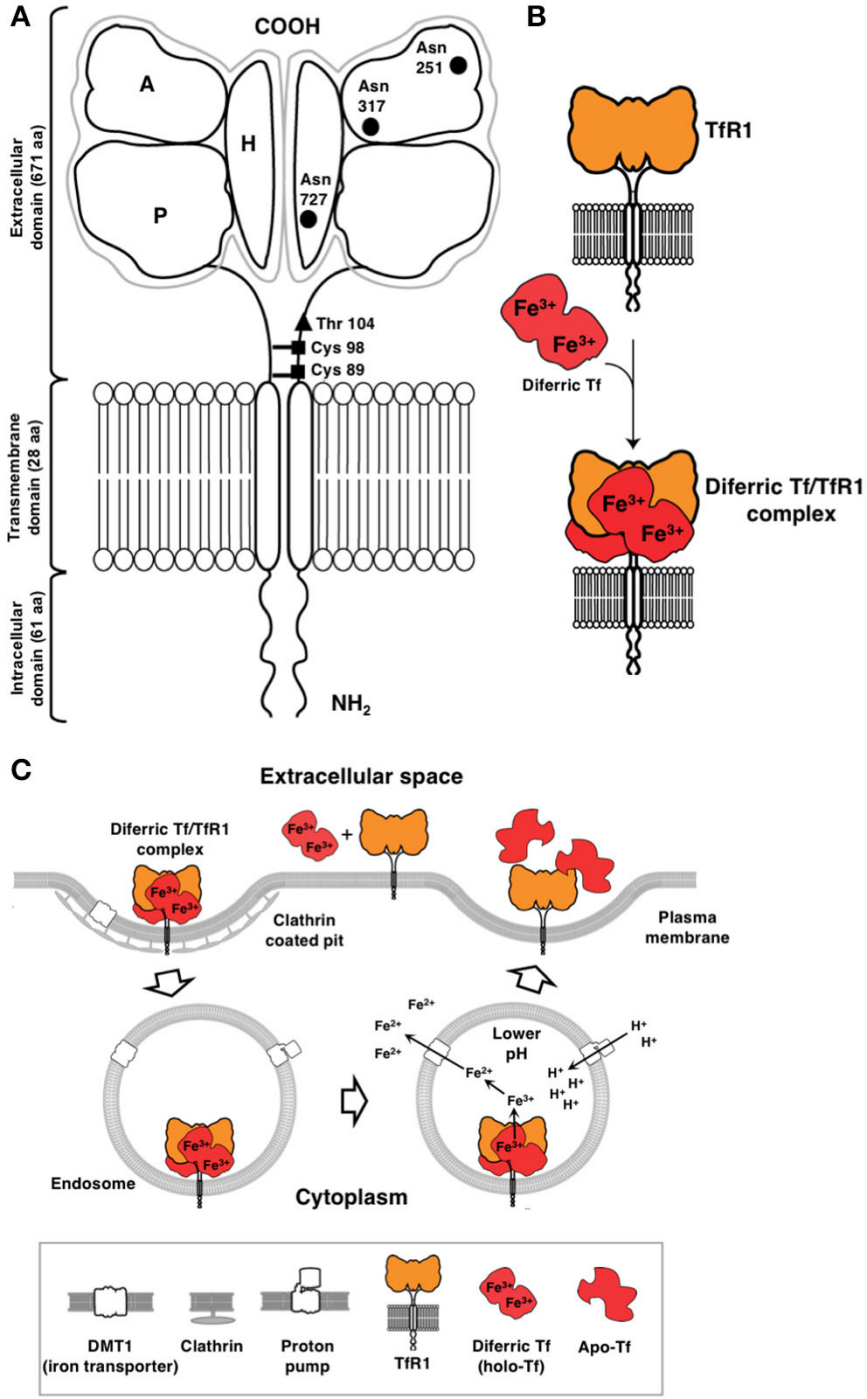
Cellular uptake of iron through the TfR1/Tf system via receptor-mediated endocytosis. **(A)** Schematic representation of TfR1. This receptor is a type II receptor found on the cell surface as a homodimer consisting of two monomers linked by disulfide bonds at cysteine (Cys) residues 89 and 98 (■). The TfR1 contains an intracellular domain, a transmembrane domain, and a large extracellular domain. There is an *O*-linked glycosylation site at threonine (Thr) 104 (▴) and three *N*-linked glycosylation sites on asparagine (Asn) residues 251, 317, and 727 (•). The extracellular domain of the TfR1 consists of three subdomains: apical (A), helical (H), and protease-like domain (P). **(B)** TfR1 consists of a dimer on the surface of the cell. Each receptor monomer binds one Tf molecule that consists of lobes (the N- and C-lobes). Each lobe binds one iron atom. Diferric Tf, also known as holo-Tf, contains two atoms of iron and binds to the receptor with high affinity. **(C)** Endocytosis of the diferric Tf/TfR1 complex occurs via clathrin-coated pits and the complex is delivered into endosomes. Protons are pumped into the endosome resulting in a decrease in pH (endosomal acidification) that triggers a conformational change in Tf and TfR1 and the subsequent release of iron. The iron is then transported out of the endosome into the cytosol by DMT1. Apo-Tf remains bound to TfR1 while in the endosome and is released once the complex reaches the cell surface. This figure was adapted and reprinted from Daniels et al. (11) with permission from Elsevier.

There are two types of transferrin receptors: TfR1, also known as cluster of differentiation 71 (CD71), which is widely expressed and binds Tf with higher affinity and the less common TfR2, which is predominantly expressed in hepatocytes (9–13). TfR1, the focus of this review, is a 90 kDa type II transmembrane glycoprotein consisting of 760 amino acids that is found as a dimer (180 kDa) linked by disulfide bonds on the cell surface (Figure 1A) (11). The TfR1 monomer is composed of a large extracellular, C-terminal domain of 671 amino acids containing the Tf binding site, a transmembrane domain (28 amino acids), and an intracellular N-terminal domain (61 amino acids). The C-terminal extracellular domain contains three *N*-linked glycosylation sites at asparagine residues 251, 317, and 727 and one *O*-linked glycosylation site at threonine 104, which are all required for adequate function of the receptor (11). Tf consists of a polypeptide chain composed of 679 amino acids and two carbohydrate chains. It is an 80 kDa glycoprotein composed of two 40 kDa subunits, known as the N- and C-lobes that are separated by a short linker sequence. Each subunit is capable of binding one free ferric iron (Fe^3+^) and thus, Tf may have up to two atoms of iron attached. Tf in its iron free form, apo-Tf, binds Fe^3+^ with high efficiency in the blood and transports it to the cell surface for internalization through the interaction with TfR1 (Figure 1B). Holo-Tf binds the “bottom” of the TfR1 close to the cell membrane, referred to as the “basal portion” that is formed by the helical and protease-like domains (14–16).

#### TfR1 Function

In terms of affinity for TfR1, diferric Tf or holo-Tf (two iron atoms bound to Tf) has the highest affinity (*K*_*d*__1_ < 0.1 nM, *K*_*d*__2_ = 3.8 nM, pH 7.4) as compared to apo-Tf (Tf lacking iron; *K*_*d*__1_ = 49 nM, *K*_*d*__2_ = 344 nM, pH 7.4) (9). Since TfR1 is found as a dimer and can bind two Tf molecules, the affinities cited above were determined by modeling the surface plasmon resonance (SPR) binding data using a heterogenous model with two independent binding sites (9). Another study reports a binding affinity of *K*_*d*_ = 4 nM for diferric Tf and a *K*_*d*_ ~30 nM for both N-lobe and C-lobe monoferric Tf, at a pH of 7.4 (17). Both of these studies are consistent with another study that reported a *K*_*d*_ of 5 × 10^−9^ M for diferric Tf and 10–100 fold less for apo-Tf (18). Thus, the receptor preferentially binds diferric Tf forming a ligand-receptor complex on the cell surface, which is constitutively internalized via clathrin-mediated endocytosis (Figure 1C) (11). In the endosome, an influx of protons mediated by an endosomal membrane proton pump leads to the lowering of the pH to 5.5 (endosomal acidification) that triggers a conformational change in both Tf and TfR1, which results in the subsequent release of iron from Tf (19). Iron is then converted from Fe^3+^ to ferrous iron (Fe^2+^) by the ferrireductase six transmembrane epithelial antigen of prostate 3 (STEAP3) enzyme and pumped out of the endosome by the divalent metal transporter 1 (DMT1) (19). The receptor/apo-Tf complex is then recycled back to the cell surface where apo-Tf is released from the receptor. Iron in the labile cytoplasmic pool is incorporated into heme and into various enzymes, serving as a co-factor to drive numerous critical cellular processes, such as those described above. Excess iron can be stored as ferritin (10–13, 18, 20). This cytoplasmic protein is a large, multi-subunit protein composed of 24 H- and L-subunits in different ratios, creating a “cage-shaped complex” (10, 21). Interestingly, H-ferritin, which is present in the blood, interacts with TfR1 through its apical domain and is internalized, providing an alternative way for iron uptake (10, 14, 22). However, TfR1 expression must be high and reach a threshold level, such as that observed in erythroblasts, for this to occur, suggesting that more than one TfR1 complex may be required for H-ferritin subunit internalization (10, 23). H-ferritin is internalized by endocytosis and transported to the lysosome for degradation (10, 22).

#### TfR1 Expression in Normal and Cancer Cells

In general, TfR1 is expressed at low levels on most normal cells (10, 11, 24, 25). Increased expression is observed on cells with a high rate of proliferation, including those of the basal epidermis, intestinal epithelium, and certain activated immune cells (11). However, high expression is also observed on cells with a high need of iron, such as placental trophoblasts and erythroid progenitors due to the high requirement of iron for heme synthesis. In addition, cells of the vascular endothelium of the brain capillaries that compose the blood-brain barrier (BBB) also express high levels of the receptor (11).

As disruption of iron homeostasis may have potentially detrimental consequences to the cell, the expression of TfR1 is tightly regulated. Normal expression of TfR1 is predominantly controlled by two iron-response element binding proteins (IRPs): IRP1, and IRP2. They bind to iron responsive elements (IREs) located on the 3′ untranslated region of the transcript of the gene for TfR1, *TFRC*. Binding of IRPs to IREs on the mRNA encoding TfR1 increases the expression of TfR1 by stabilizing the transcript (11, 26–28). Other genes also influence TfR1 expression, with most of them having a role in cancer pathogenesis. The proto-oncogene *c-MYC* encodes the oncogenic transcription factor c-MYC, which has been shown to directly regulate the expression of *TFRC* via binding to a conserved E box binding site in intron 1 of *TFRC* (29). Hypoxia inducible factor 1 (HIF-1), which consists of the protein subunits encoded by the two genes (*HIF1A* and *HIF1B*), is an important DNA-binding protein involved in many aspects of cancer pathogenesis including metabolic reprogramming, inflammation, angiogenesis, and resistance to therapeutics (30–33). HIF-1 activates the expression of *TFRC* in iron deficient conditions via binding to an upstream hypoxia response element (34). In breast cancer, the oncogene *SRC* encodes the tyrosine kinase Src that phosphorylates the tyrosine 20 residue in the cytoplasmic region of TfR1 and potentiates breast cancer cell survival and inhibits apoptosis (35). Expression of TfR1 is also enhanced by the loss of the gene encoding sirtuin 3 (*SIRT3*), a mitochondrial deacetylase (36). Loss of *SIRT3* increases the production of reactive oxygen species (ROS) leading to increased IRP1 binding to IREs, promoting *TFRC* transcription. TfR1 expression is also increased by the sex hormone and growth factor 17β-estradiol in estrogen receptor positive breast cancer (37). Overall, TfR1 expression is influenced by numerous genes and growth factors, many of which are pro-tumorigenic and/or promote the formation of aggressive malignancies. Many of these factors regulating the expression of TfR1 coalesce around or contribute to the classic TfR1 role as a mediator of cellular proliferation. Unregulated and uncontrolled proliferation of malignant cells is one of the hallmarks of cancer (38, 39). Increased iron uptake through TfR1-mediated endocytosis provides iron to power this replicative biosynthetic machinery (1). Due to the high rate of proliferation of most cancer cells, TfR1 is overexpressed on malignant cells (11, 24, 25, 40–77) at least in part due to the high activity of the ribonucleotide reductase enzyme that requires iron as a cofactor and is needed for DNA synthesis and cellular proliferation, as mentioned above. It has also been noted that malignant cells are more dependent on iron for growth compared to normal cells, a phenomenon known as “iron addiction” (3, 59). Thus, cancer cells are more sensitive to iron deprivation.

In addition, intracellular iron protects cancer cells against natural killer (NK) cells (78) and it has also been demonstrated that the heavy chain of the iron-storing protein ferritin inhibits apoptosis induced by tumor necrosis factor alpha (TNFα) through the suppression of ROS accumulation (79). TfR1 has also been shown to mediate NF-κB signaling in malignant cells through the interaction with the inhibitor of the NF-κB kinase (IKK) complex, increasing cancer cell survival (80). Of interest, NF-κB can also induce TfR1 expression via regulation of HIF-1α levels (81–83), establishing another connection between these molecules. TfR1 can also contribute to the modulation of mitochondrial respiration and the production of mitochondria-derived ROS, which play crucial roles in malignant cell growth and survival (84). Interestingly, TfR1 has shown a potential role in maintaining the stemness of hepatocellular carcinoma-derived cancer stem-like cells and promoting malignant behavior by regulating iron accumulation in these cells (85).

Due to the aforementioned reasons, TfR1 is overexpressed on many different types of cancer cells, often at levels several-fold higher than normal cells (11, 24, 25, 40–77). In fact, TfR1 has been identified as a universal cancer marker (55). Increased expression of TfR1 correlates with advanced stage and/or poorer prognosis in a number of cancers, including solid cancers such as esophageal squamous cell carcinoma (56), breast cancer (57, 58), ovarian cancer (59), lung cancer (60), cervical cancer (61), bladder cancer (62), osteosarcoma (63), pancreatic cancers (64), cholangiocarcinoma (65), renal cell carcinoma (66), hepatocellular carcinoma (67, 68), adrenal cortical carcinoma (69), and cancers of the nervous system (70) as well as hematopoietic malignancies such as acute lymphoblastic leukemia (ALL) (71, 72), chronic lymphocytic leukemia (CLL) (73), and non-Hodgkin lymphoma (NHL) (73, 74). Interestingly, patients infected with the human immunodeficiency virus (HIV) often develop more aggressive NHL that have been shown to express even higher levels of TfR1 messenger RNA compared to NHL cells from non-infected patients (75, 76).

## Targeting TfR1 for Cancer Therapy

The elevated levels of TfR1 expression on malignant cells, together with its extracellular accessibility, ability to internalize, and central role in cancer cell pathology make this receptor an attractive target for antibody-mediated therapy. TfR1 can be targeted for cancer therapy in two distinct ways. One way widely used is indirectly through the use of Tf, ferritin, or antibodies specific for TfR1 for the purpose of anti-cancer drug delivery, including the delivery of chemotherapeutics, proteins such as toxins, nucleic acids such as oligonucleotides, and viral vectors (86–89). Nanodrugs and other formulations, such as antibody drug conjugates (ADC) or probody drug conjugates (PDC) that target TfR1 can also be produced (86–90). Targeting TfR1 for this purpose has been extensively reviewed (86–89) and is not the focus of this article. The other way is to use the antibodies themselves as anti-cancer agents, utilizing their ability to directly inhibit TfR1 function and/or activate antibody-mediated effector functions such as antibody-dependent cell-mediated cytotoxicity (ADCC), antibody-dependent cell-mediated phagocytosis (ADCP), and complement-dependent cytotoxicity (CDC). Importantly, the two ways TfR1 can be targeted for cancer therapy are not mutually exclusive. Antibodies that are directly cytotoxic may also be used for delivery purposes. However, this article focuses on antibodies targeting TfR1 that are used as direct anti-cancer agents.

**Figure 2:**
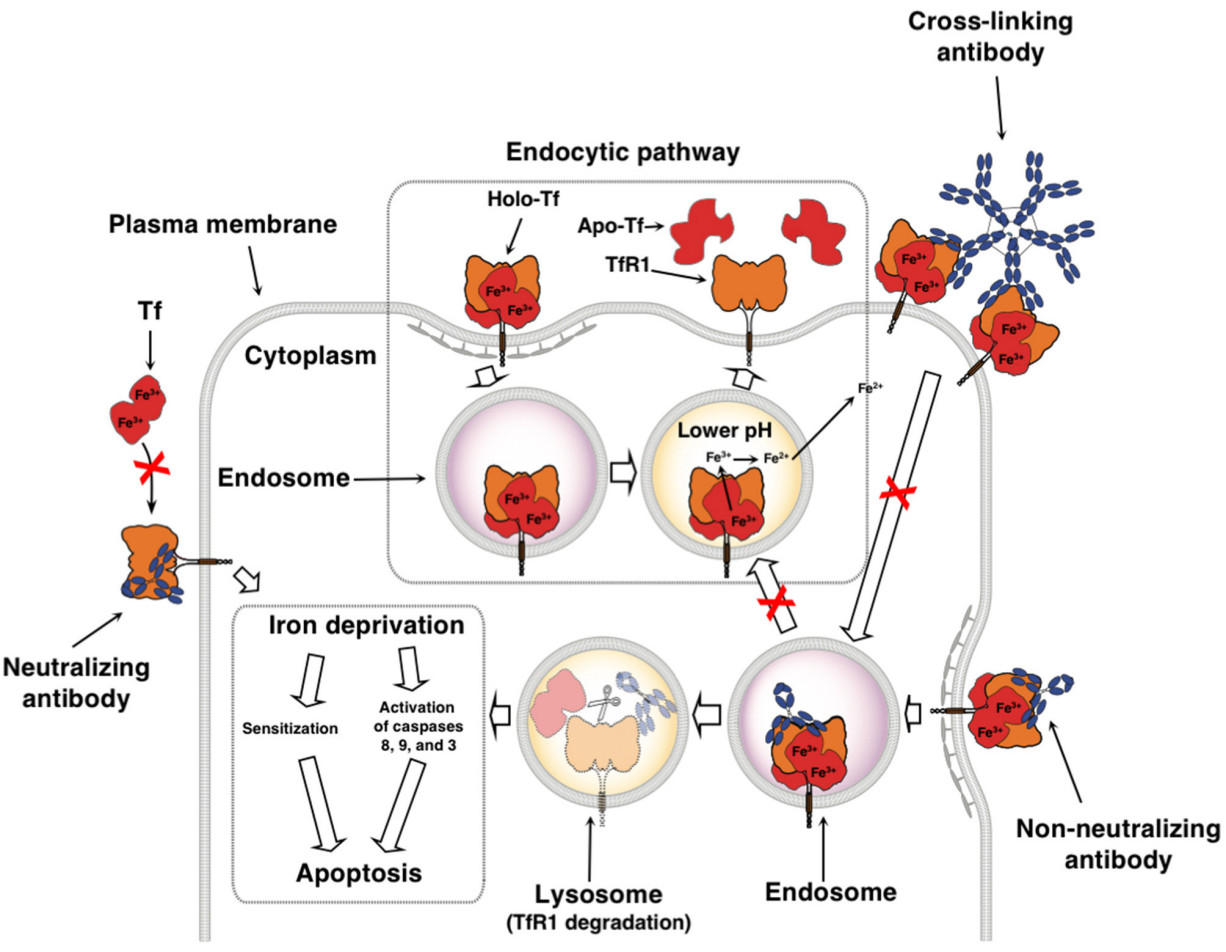
Therapeutic antibodies targeting TfR1 can disrupt iron uptake through multiple non-mutally exclusive mechanisms. Neutralizing antibodies inhibit Tf from binding to TfR1, blocking the uptake of iron. Non-neutralizing antibodies still allow the binding of Tf, but may disrupt TfR1 cycling and induce degradation of TfR1. Binding of multivalent antibodies, such as IgM, to TfR1 may result in extensive cross-linking of the receptor and inhibition of its internalization. All of these ultimately lead to iron deprivation that can either sensitize cells to other anti-cancer agents or can cause the inhibition of proliferation and induction of caspase activation resulting in apoptosis. This figure was adapted and reprinted with permission from Springer Nature: Humana Press, Inc., Daniels et al. Targeting the transferrin receptor to overcome resistance to anti-cancer agents. *In* Sensitization of Cancer Cells to Chemo/Immuno/RadioTherapy (91).

**Figure 3:**
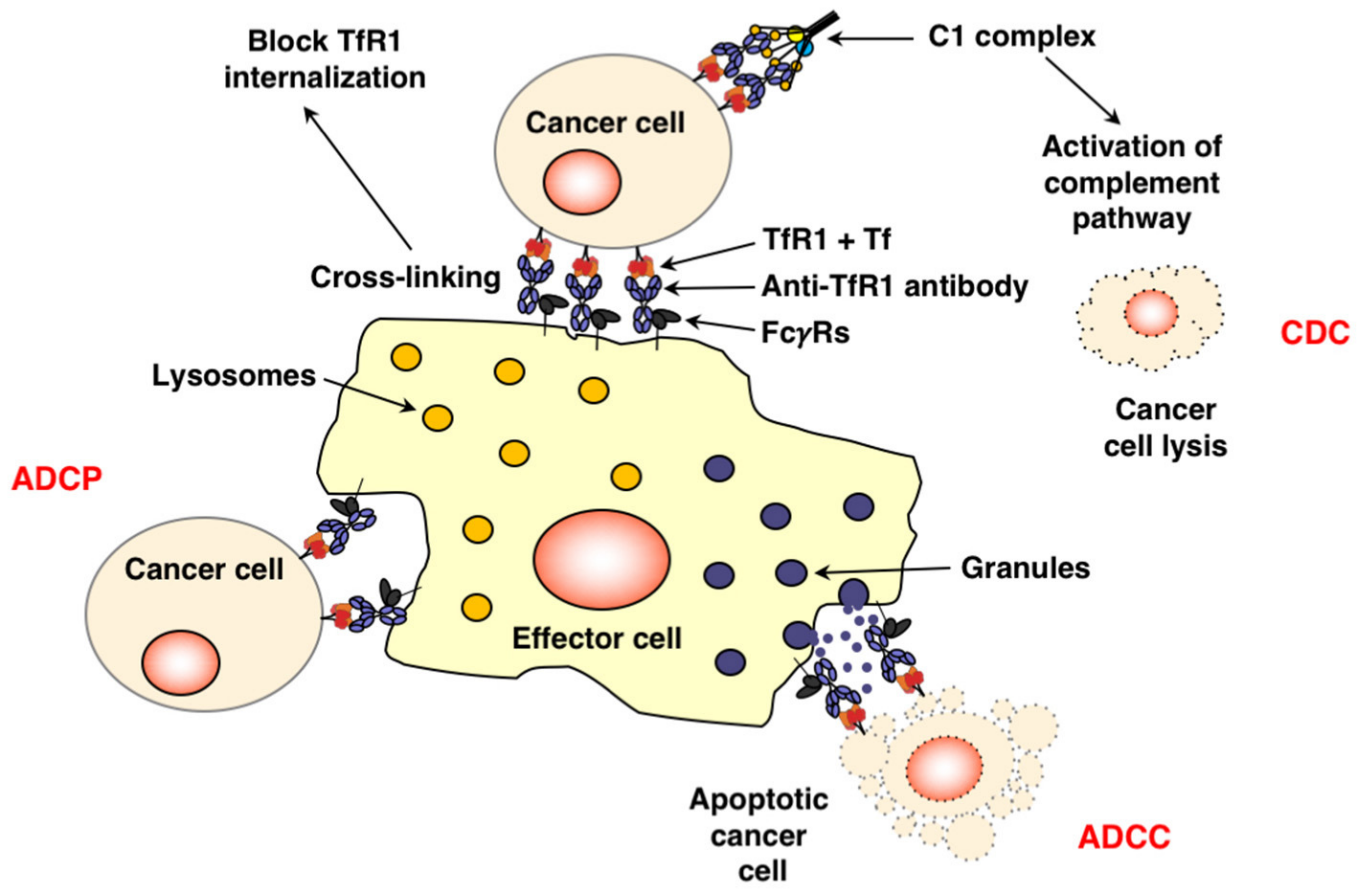
Fc-mediated functions of anti-TfR1 antibodies. The simultaneous binding of an antibody, such as IgG1, to TfR1 on the surface of the cancer cell and the FcγRs on the surface of effector cells through the Fc region may induce cell death through ADCC and/or ADCP. This interaction may also potentially sequester TfR1 on the surface of the cell, inhibiting its internalization. Antibodies bound to apoptotic cells or apoptotic bodies are also expected to trigger phagocytosis. Additionally, binding of the antibody to the antigen on the surface of the cancer and to C1q, the first component of the classical complement pathway, through the antibody Fc region may also lead to cell lysis through CDC.

As stated above, cancer cells have an increased requirement for TfR1 and iron for multiple functions. The successful inhibition of TfR1 function by anti-TfR1 antibodies leads to iron deprivation and subsequently the death of cancer cells (11, 91). Depending on the location of the targeted epitope, antibodies can interfere with TfR1 function through various mechanisms (Figure 2). Anti-TfR1 antibodies that inhibit the binding of Tf to the receptor are known as neutralizing antibodies and block iron-loaded Tf uptake. A non-neutralizing antibody, which does not interfere with Tf binding to TfR1, can also indirectly inhibit iron intake by preventing the internalization of the receptor and thus, iron-loaded Tf, through extensive cross-linking of TfR1 on the cell surface (92, 93). Another possibility is that TfR1 internalizes with the bound anti-TfR1 antibody, resulting in the impaired recycling of the receptor and its degradation in the lysosome with the subsequent decrease of cell surface TfR1 (92–96). All of these methods ultimately block iron uptake, leading to impaired TR1 function and lethal iron deprivation. There are three antibody-mediated effector functions that can play crucial anti-cancer roles: ADCC, ADCP, and CDC (Figure 3) (97–99). These functions are mediated by the Fc region of the antibody, which binds Fc gamma receptors (FcγRs) on the surface of immune effector cells triggering a variety of possible anti-tumor actions depending on the effector cell engaged. Upon binding to the FcγRs on NK cells, the antibody mediates ADCC, inducing the release of cytolytic granules and cytokines that may further enhance the anti-tumor response (100). Other effector cells capable of triggering ADCC are neutrophils and macrophages (101). Importantly, antibody binding to the FcγRs on the surface of macrophages facilitates ADCP, engulfment of the tumor cell in phagosomes leading to the eventual breakdown of the cancer cell (102). In addition, as macrophages are also antigen-presenting cells (APCs), they can improve anti-tumor immunity through the possible induction of a secondary immune response against processed and presented tumor antigens (103, 104). Furthermore, the simultaneous interaction of the antibody with FcγRs on the surface of immune cells and TfR1 on targeted cancer cells may also potentially sequester the receptor on the surface of the cell, inhibiting its internalization and iron uptake. Moreover, the Fc region of antibodies can also induce CDC by activating the C1q/C1 complex of the classical complement cascade with the eventual assembly of the membrane attack complex on the cancer cell, leading to cell death (105). Advances in genetic engineering have resulted in antibodies that may have increased interaction with these immune mechanisms (97, 98).

### Anti-TfR1 Antibodies

#### Antibody Structure, Classes, and Engineered Formats

**Figure 4:**
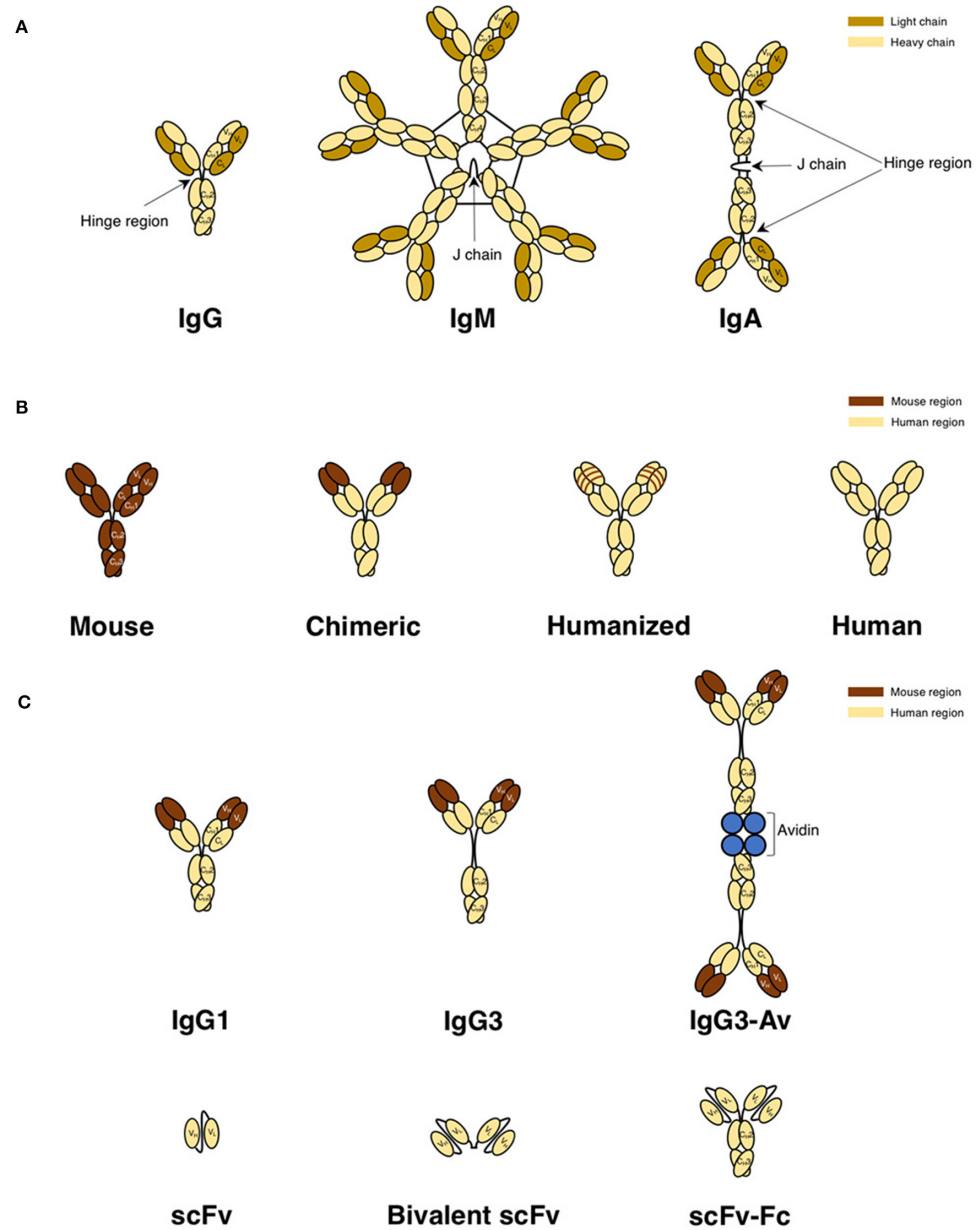
Antibody classes and derivatives used to target TfR1 for cancer therapy. **(A)** Naturally occurring antibody formats IgG, IgM, and IgA in mice, rats, and humans. IgG, representing the basic structure of an antibody, is composed of two light and two heavy chains. The light chain is composed of variable light (V_L_) and constant light (C_L_) domains. The heavy chain is composed of one variable heavy (V_H_) domain and three constant heavy domains (C_H_1, C_H_2, and C_H_3) and contains a hinge region between the C_H_1 and C_H_2 domains that provides flexibility to the molecule. IgM is a pentamer with a joining (J) chain, but can also be found as a hexamer without a J chain (not shown). IgA is a dimer with a J chain. **(B)** Representations of a mouse IgG antibody, a mouse/human chimeric antibody with murine variable regions, a CDR-grafted or “humanized” antibody with murine CDRs, and a fully human antibody. **(C)** Structurally modified human antibody formats. Both the IgG1 and IgG3 are chimeric antibodies that have murine variable regions and human constant regions, while the avidin fusion protein is the chimeric IgG3 with chicken avidin genetically fused to the C-terminus of each heavy chain. Due to the tetrameric structure of avidin, this fusion protein exists as a dimer in solution. The three smaller antibody formats (scFv, divalent scFv, and scFv genetically fused to the Fc fragment) contain all human domains.

Antibodies consist of two light and two heavy chains in a heterodimeric structure held together by disulfide bonds (98, 106). The variable regions bind specifically to the antigen, while the Fc region mediates effector functions and is responsible for the pharmacokinetic properties of the antibody. The antibody classes discussed in this review are shown in Figure 4A and are from human, mouse, or rat origin. Most antibodies have a hinge region that is located between the first (C_H_1) and second (C_H_2) constant domains of the heavy chain. However, IgM antibodies lack this hinge region and instead have an extra constant domain in the μ heavy chain. Thus, IgM has four heavy chain constant domains compared to three heavy chain constant domains in IgG antibodies. IgM exists as a pentamer with a joining (J) chain, a polypeptide that interacts with the cysteine in the tailpiece to promote polymerization. Thus, pentameric IgM has a molecular weight (m.w.) of ~970 kDa. IgM can also be found as a hexamer lacking the J chain. In contrast, the IgA antibody (~390 kDa m.w.) is dimeric in structure and is joined by a J chain (98, 106). IgG antibodies are monomeric. Human IgG antibodies are further divided into four subclasses: IgG1, IgG2, IgG3, and IgG4. Human IgG3 is unique in that it has an extended hinge region making the m.w. higher (~165 kDa) compared to the other human IgG subclasses (~150 kDa). Mouse IgG antibodies are similarly divided into four subclasses: IgG1, IgG2a, IgG2b, and IgG3. However, there are no direct homologs between human and mouse IgG subclasses. Mouse IgG2a and human IgG1 are typically considered to be functionally equivalent, while mouse IgG1 and human IgG4 are close functional analogs due to the lack of binding to the activating FcγRs and inability to induce Fc-mediated effector functions (107–109). Rat IgG antibodies are also divided into four subclasses: IgG1, IgG2a, IgG2b, and IgG2c. Rat IgG2a and IgG1 are considered to be functionally equivalent to mouse IgG1, while rat IgG2b is equivalent to mouse IgG2a, and rat IgG2c is equivalent to mouse IgG3 (110, 111). Rat IgG2b is most effective at activating complement and inducing ADCC (111).

Since the Fc region of murine antibodies are immunogenic in humans and interact poorly with human effector cells, antibodies targeting TfR1 with human constant regions have been developed (Figure 4B) with the goal that they are better tolerated, interact with the immune system and thus, result in higher efficacy in human patients. The first step to decreasing immunogenicity of murine antibodies was through the use of chimeric antibodies, or antibodies composed of sequences from two different species. Mouse/human chimeric antibodies have murine variable regions but contain human constant regions and are a step toward therapeutic antibodies that are better tolerated and have proper interaction with host effector mechanisms compared to antibodies from non-human origins (97, 98, 112). A humanized antibody contains the complementarity-determining regions (CDRs) within the variable regions are of murine origin while its framework regions (FRs) are of human origin (97, 98).

Importantly, antibodies can be further modified through genetic engineering (98, 106). Mouse/human chimeric antibodies can exist as any antibody subclass. For example, the anti-TfR1 antibody ch128.1, discussed below, was produced as both IgG3 and IgG1 antibodies. Additionally, avidin can be added to the C-terminus of the heavy chain to produce a fusion protein (Figure 4C). A single chain Fv (scFv) is the smallest functional antigen-binding unit, expressed as a single polypeptide of ~25 kDa m.w., and is composed of the variable domains of the heavy and light chains (V_L_ and V_H_) that are genetically linked together through an artificial flexible linker (Figure 4C). Due to their small size, these fragments show a high level of tumor targeting and are better suited to penetrate solid tumors compared to full-length antibodies. They can also be expressed in low-cost, prokaryotic systems due to the lack of glycosylation and simple structure. However, since scFv fragments lack Fc regions, they do not bind the neonatal receptor (FcRn), also known as the Brambell or “salvage receptor,” that largely determines the long half-life in blood (bioavailablity) of antibodies. Thus, scFv fragments have much shorter half-lives in blood compared to full length antibodies and are rapidly cleared from the circulation (98, 106). This limits their bioavailability and decreases anti-tumor efficacy. scFv are also not able to stimulate the Fc-mediated effector functions so the anti-tumor activity of these antibody fragments is limited to the direct interference with TfR1 function. An additional limitation of scFv is that they are monovalent. Full length antibodies demonstrate higher avidity due to the presence of two antigen-binding sites. To overcome this limitation, dimers of scFv (known as “diabodies” or bivalent scFv) that have two antigen-binding sites can be engineered by adding a cysteine to the C-terminus of the scFv to form a disulfide bond between the two scFvs (Figure 4C) (98, 106). However, these bivalent scFv do not contain an Fc region and thus, are still incapable of eliciting antibody effector functions. Engineering scFv-Fc molecules that contain two scFv genetically fused to the C_H_2 and C_H_3 domains of the Fc region overcomes this limitation as well as the inability to bind the FcRn (Figure 4C) (98, 106).

There are a number of anti-TfR1 antibodies and/or antibody derivatives that show direct anti-cancer activity (Supplementary Table 1). This article reviews these antibodies and their anti-cancer effects.

#### Rat Anti-mouse TfR1 Antibodies

The earliest monoclonal antibodies were developed using the classical hybridoma technology (113). Antibodies RI7 208, REM 17.2, and RI7 217 were generated using this method (92, 114, 115). RI7 208 is a non-neutralizing rat anti-mouse TfR1 IgM monoclonal antibody that inhibits iron uptake and *in vitro* proliferation of murine myeloma S194/5.XXO.BU.1 cells (114, 115). This IgM antibody also inhibits iron uptake and the *in vitro* proliferation of AKR1 murine lymphoma cells due to extensive cross-linking of TfR1 receptors on the cell surface, preventing endocytosis of the Tf/TfR1 complex (92, 114, 115). RI7 208 also prolongs the *in vivo* survival of AKR/J mice bearing murine SL-2 leukemic cells in a syngeneic tumor model (116). Acute toxicity in treated mice was minimal with no gross toxicity observed, and histological examinations of small intestine, liver, and spleen found no evidence of cell level damage (116). However, there were significant differences in erythroid progenitor numbers in the bone marrow and the spleens of the mice, with a two-fold decrease in erythroid progenitors in the bone marrow and a three-fold increase in the spleen of antibody treated mice as compared with control mice. Levels of myeloid progenitors in the bone marrow and spleen followed the same trend as erythroid progenitors though the relative differences between treated and control were smaller (116).

The other rat anti-mouse TfR1 IgM antibody REM 17.2, also a non-neutralizing antibody, prevents iron intake by extensive cross-linking of cell surface TfR1. It inhibits cancer cell proliferation *in vitro* and elicits CDC in the presence of rabbit complement, but did not prolong the survival of mice bearing SL2 leukemic cells in the syngeneic tumor model mentioned above (92, 116). RI7 217 is a non-neutralizing rat anti-mouse TfR1 IgG2a antibody that decreased cell surface TfR1 levels, increased TfR1 degradation, and decreased cell growth rates (92). The addition of a secondary anti-mouse IgG antibody enhanced this toxicity and mimicked the growth inhibitory effects of the IgM antibodies (92). Therefore, cross-linking of cell surface TfR1 is vital for the inhibitory effects of RI7 208 and REM 17.2. The RI7 217 antibody has also been used for delivery purposes in cancer therapy (86, 87).

#### Murine Anti-chicken TfR1 Antibodies

Antibodies specific for chicken TfR1 have also been evaluated for intrinsic cytotoxic activity against cancer cells. D18 and D19 are non-neutralizing mouse IgG2a antibodies targeting the chicken TfR1 (117, 118). These antibodies inhibit the *in vitro* proliferation and increased cell death of DT40 chicken B-lymphoid cancer cells (117, 118). The inability of z-VAD-fmk, a pan-caspase inhibitor, to rescue this effect indicates that caspase activation is not essential to inducing cell death in this model (117). In addition, cell death induced by these antibodies had characteristics of autophagic (cytoplasmic vacuoles) and necrosis-like (ruptured plasma membranes) cell death (117), leading the authors to suggest that the TfR1 may be acting as a death receptor (117, 118).

#### Murine Anti-human TfR1 Antibodies

Multiple murine anti-human TfR1 antibodies have been described in the literature. Many of them do not have intrinsic anti-proliferative/pro-apoptotic effects on cancer cells. Therefore, only those that have shown cytotoxic effects are described in this section. Examples of these antibodies are 7579, B3/25, 43/31, D65.30, A24, RBC4, and 42/6 (94, 119–134).

The murine anti-human TfR1 IgG antibody 7579 downregulates TfR1 surface levels on cancer cells (132) and has shown efficacy in mitigating *in vitro* proliferation as well as inducing apoptosis of human U87MG, U251, and A172 glioma cells, human HepG2 hepatoma cells, and human MCF7 breast cancer cells (120–122, 132). When used in combination with curcumin, a polyphenolic compound found in the spice turmeric, which can act as an iron chelator, a synergistic effect on the induction of a necrotic form of cell death in glioma cells was observed (120). A synergistic cytotoxic effect in glioma cells was also observed when 7579 was combined with the chemotherapeutic drug nimustine (122). In addition, a synergistic cytotoxic effect was observed when 7579 was combined with the chemotherapeutic 5-fluorouracil, while an additive effect was observed when it was combined with the chemotherapeutic doxorubicin in MCF7 and HepG2 cells (132). 7579 also synergizes with sinomenine hydrochloride, an anti-inflammatory plant alkaloid used to treat autoimmune diseases, to greatly inhibit proliferation and promote apoptosis in HepG2 cells, which was dependent on the COX-2 pathway (121). The 7579 antibody has also been used as a fusion protein for delivery of an anti-cancer agent (86).

E2.3 and A27.15, both murine IgG1 antibodies, have shown minor or inconsistent anti-tumor effects as single agents. However, when used in combination, these antibodies have shown robust anti-cancer effects (125, 133). E2.3 in combination with A27.15, a neutralizing antibody, shows anti-tumor activity in U266 human multiple myeloma (MM) cells *in vitro* with equal amounts of each antibody together decreasing the proliferation rate of U266 cells (125). In MDA-MB-468, MDA-MB-231, and MCF-7 human breast cancer cell lines, the combination of E2.3 and A27.15 inhibits cellular proliferation *in vitro* (133).

B3/25 is a non-neutralizing murine anti-human TfR1 IgG1 antibody and 43/31 is a neutralizing murine anti-human IgG1 antibody, both of which have demonstrated anti-cancer effects (123, 126, 129). Both B3/25 and 43/31 have been shown to inhibit the *in vitro* proliferation of the human myeloid leukemia cell lines HL-60 and KG-1 as well as inhibiting colony forming units—granulocyte/macrophage (CFU-GM), and thus, normal hematopoietic cell differentiation (126). B3/25 has been used for the delivery of toxins into cancer cells (86, 87). D65.30 is a non-neutralizing murine anti-human TfR1 IgG1 antibody that inhibits *in vitro* HL-60 leukemia colony formation and human ALL (T-cell origin) CCRF-CEM cell proliferation (127). D65.30 also demonstrated limited anti-tumor efficacy *in vivo* against CCRF-CEM subcutaneous (s.c.) tumor growth in xenograft murine models (127). However, treatment of these mice with D65.30 combined with the A27.15 antibody (described above) resulted in a relevant anti-tumor growth effect (127).

**Figure 5:**
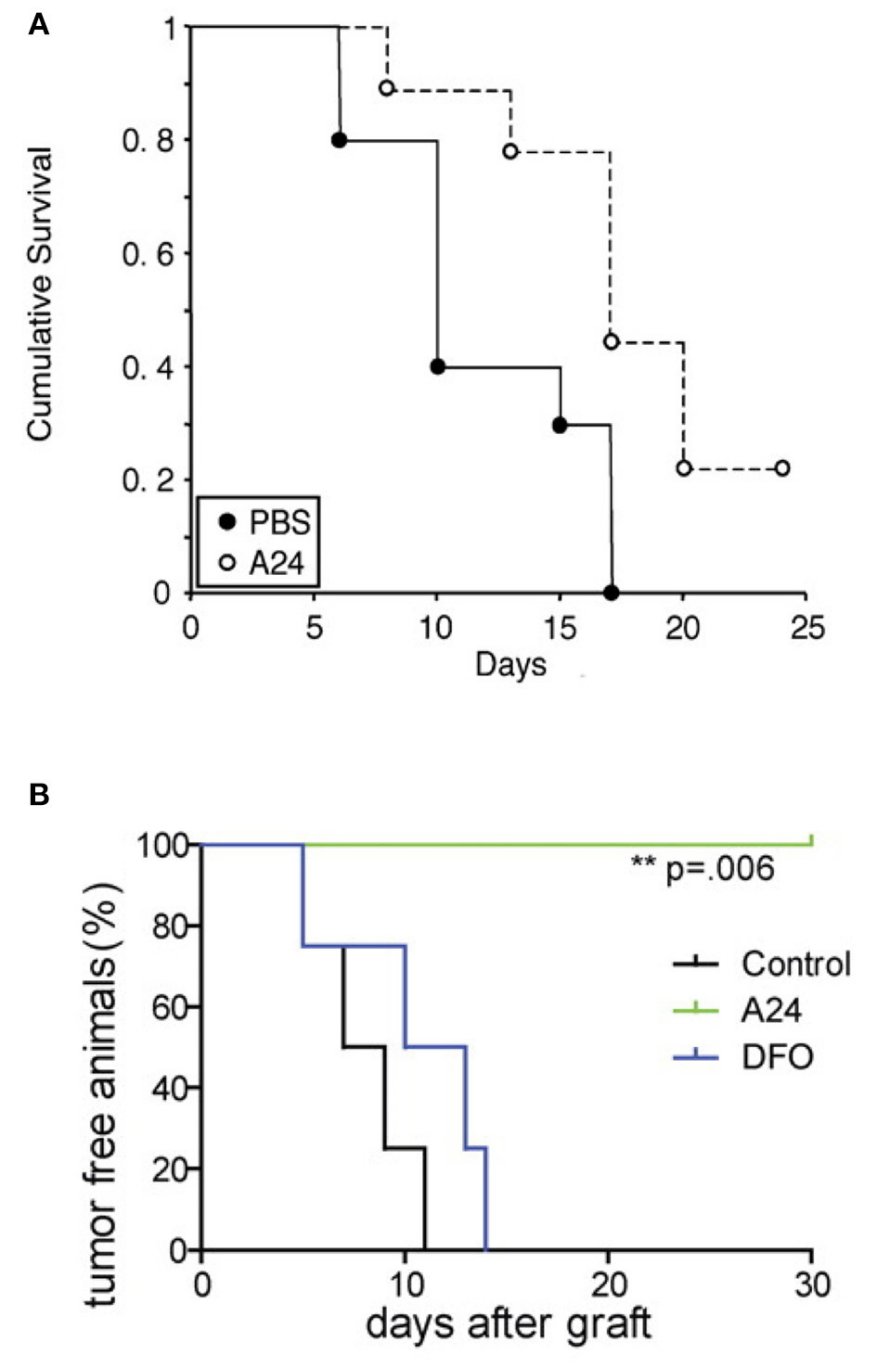
Anti-tumor activity of the murine IgG2b antibody A24 in xenograft mouse models. **(A)** Kaplan–Meier survival curve of athymic nude mice bearing s.c. MCL UPN1 tumors treated with a single (i.v.) dose of 40 mg/kg A24 (10 mice) or vehicle control (9 mice). Treatment was initiated when tumors were about 5 mm in diameter. This figure was reprinted by permission from the American Association for Cancer Research: Lepelletier et al. (119). **(B)** Kaplan–Meier survival curve of the percentage of tumor-free mice among mice treated with vehicle control, A24 (single i.p. dose of 40 mg/kg), or DFO (20 mg 5 times per week) after s.c. xenograft with AML HL60 cells (*n* = 4 in each group). The *p*-value was determined using the log-rank test. One representative experiment of three is shown. © 2010 Callens et al. (128).

A24 is a neutralizing murine anti-human TfR1 IgG2b antibody that has been shown to inhibit the *in vitro* proliferation and induce apoptosis in adult T-cell leukemia/lymphoma (ATLL), acute myeloid leukemia (AML), and mantle cell lymphoma (MCL) cells (94, 119, 128). Its mechanism of action includes the sequestration and degradation of TfR1 via rerouting endosomes containing antibody-bound TfR1 from the normal TfR1 cycling process to fuse with lysosomal compartments where it is degraded (94, 119). This results in decreased receptor levels, concomitant decreased iron uptake, which in turn induces iron deprivation and leads to apoptosis (94, 119). *In vitro*, A24 also inhibited the proliferation of primary AML blasts (128). A24 has also shown significant anti-tumor activity *in vivo* (119, 128). In a xenograft mouse model of human MCL using the UPN1 cell line and athymic nude mice, intravenous (i.v.) treatment with A24 at a single dose of 40 mg/kg prevented s.c. tumor establishment (119). This same dose of A24 delayed established UPN1 tumor growth and prolonged mouse survival in a treatment-delayed tumor growth model, where treatment was initiated when tumors reached an approximate diameter of 5 mm (Figure 5A) (119). In another mouse xenograft model with s.c. HL-60 tumors, intraperitoneal (i.p.) treatment with A24 1 day after tumor inoculation is sufficient to prevent tumor establishment as compared with vehicle control or deferoxamine (DFO), an iron chelator (Figure 5B) (128). The superior activity of A24 compared to the iron chelator may be explained, at least in part, by the contribution of antibody effector functions such as ADCC and CDC, the ability of the antibody to induce specific TfR1 downregulation in cancer cells expressing high TfR1 levels, and the possibility that the iron chelator induces TfR1 messenger RNA stability through a post-transcriptional mechanism (128, 134).

Other murine antibody classes apart from IgG have also been evaluated. RBC4 is a murine anti-human TfR1 IgM antibody that has been shown to inhibit the proliferation and induce cell death *in vitro* in a range of hematological cancer cell lines, such as human chronic myelogenous leukemias (CML) RPMI 8866 and UC7296 cells, as well as human Jurkat acute T-cell leukemia cells, MOLT-4 ALL (T-cell origin) cells, and HL-60 acute promyelocytic leukemia cells (APL), a subtype of AML (135). RBC4 was also shown to inhibit the proliferation of the mitogen phytohemagglutinin (PHA)-stimulated peripheral blood lymphocytes but did not affect unstimulated lymphocytes (135), consistent with the notion that activated lymphocytes express higher levels of TfR1 and are thus, more sensitive to blockage of TfR1 function.

42/6 is a murine anti-human TfR1 IgA antibody that non-competitively inhibits Tf binding to TfR1, decreases the level of TfR1 on the surface of cells, and inhibits CCRF-CEM cell proliferation *in vitro* (130). In addition, 42/6 has also shown to inhibit CFU-GM growth as well as a range of human myeloid leukemias, such as KG-1 and HL-60, although cancer cells were generally shown to be more sensitive (124, 126, 127). 42/6 has also been tested in solid tumor cell lines, such as human melanoma cell lines 242 and 354, the human ovarian cancer cell line 547, and epidermoid carcinoma A431, but inhibition of proliferation was only clearly observed in 242 cells (136). The murine IgA anti-human TfR1 antibody 42/6 had shown particularly potent cytotoxic effects against human malignant cells compared to other anti-TfR1 antibodies at that time. Results of a Phase I clinical trial in 27 patients show 42/6 was well-tolerated in general (131). Participants with different types of advanced refractory cancers received 33 treatments of 42/6 administered as 24-h infusions of 2.5 to 300 mg/m^2^. Three patients who had hematological cancers, one follicular small cleaved cell lymphoma, one Hodgkin lymphoma (HL), and one CLL, showed mixed anti-tumor responses after treatment (131). Out of 27 participants, nine patients developed human anti-mouse antibody (HAMA) responses and one patient receiving a second dose experienced an allergic type immune response associated with the development of the HAMA response (131). The HAMA response, together with the rapid clearance of murine IgA in patients could explain the lack of efficacy of 42/6. This antibody has also been utilized for drug delivery to cancer cells (86, 87).

In summary, several anti-human TfR1 antibodies of murine origin were developed early on and have been shown to be effective in inhibiting cancer cell proliferation and inducing cancer cell death by blocking TfR1 function and possibly through induction of antibody-mediated effector functions. Some have shown efficacy *in vivo* and one was well-tolerated in a clinical trial. Some of these murine anti-human TfR1 antibodies have shown enhanced anti-cancer activity in combination with other compounds. However, the use of these murine antibodies in the treatment of human cancer poses its own set of challenges. Murine antibodies do not properly interact with components of the human immune system restricting their efficacy and have a tendency to induce a HAMA response when administered into humans, which not only impacts the bioavailability of the antibody, but may also risk an allergic immune response (97, 98, 137, 138).

#### Mouse/Human Chimeric Anti-human/rat TfR1 Antibodies

To overcome the limitations of murine antibodies, a mouse/human chimeric IgG1 antibody containing the variable regions of the murine 7579 anti-human TfR1 IgG antibody was constructed with human constant regions (139). This antibody (named D2C) was shown to induce apoptosis in human K562 erythroleukemic cells through the intrinsic apoptotic pathway (140). D2C also showed the ability to mediate ADCC and CDC *in vitro* against human cancer cell lines CEM (ALL T-cell origin also known as CCRF-CEM), K562 (erythroleukemia), and SMMC-7721 (hepatocellular cancer) and *in vivo* targeting of cancer cells in a xenograft SMMC-7721 hepatocellular cancer model using nude mice (140). This antibody was also used in a combination therapy with the iron chelator curcumin. Curcumin combined with D2C showed enhanced anti-cancer effects in human PC-3 castration-resistant prostate cancer cells (141).

Our group has developed a mouse/human chimeric antibody that is composed of human IgG3 constant regions and the variable regions from the murine IgG1 antibody 128.1 (127), named ch128.1 and also known as anti-hTfR1 IgG3 or ch128.1/IgG3 (96, 142–144). This antibody was used to construct a fusion protein by inserting chicken avidin at the C-terminus of the C_H_3 constant domains of the IgG3 antibody (96, 145). The antibody-avidin fusion protein (ch128.1Av) and its parental antibody (ch128.1) are non-neutralizing antibodies as they do not interfere with Tf binding to TfR1 (96, 146). It binds to amino acid residues between serine 324 and serine 368 in the apical domain of TfR1 (147). The ch128.1Av antibody fusion protein was originally developed and has been used as a universal delivery system for biotinylated anti-tumor agents, including biotinylated lentiviral vectors and the plant toxin saporin (145, 148–151). Surprisingly, ch128.1Av was also found to have intrinsic anti-proliferative/pro-apoptotic activity *in vitro* against several human hematopoietic cancer cells, including MM cells (KMS-11, MM.1S, 8226/S, OCI-My5, and primary MM cells isolated from patients), APL cells (HL-60), Burkitt lymphoma (BL) cells (Ramos, Raji, and HS-Sultan), B-lymphoblastoid cell lines (ARH-77 and IM-9), TCL (Jurkat), and erythroleukemic cells (K562) (96, 152). These cytotoxic effects could be enhanced through the combination with other anti-cancer agents including gambogic acid (a xanthone isolated from a plant used in traditional Chinese medicine), the HXR9 peptide that prevents *HOX* gene transcription, and the chemotherapeutic cisplatin (152–154). Interestingly, targeting the TfR1 can also be a strategy to overcome resistance to anti-cancer agents (91). In fact, treatment of malignant B cells resistant to cisplatin with ch128.1Av lead to the inhibition of the NF-κB and AKT pathways, resulting in the resensitization of these malignant cells to the cytotoxic effects of cisplatin (154).

The parental antibody, ch128.1 also shows *in vitro* cytotoxic activity, although to a lesser extent than ch128.1Av. This *in vitro* cytotoxicity results from the alteration of the TfR1 cycling pathway, in which the TfR1 is routed to the lysosome where it is degraded. Thus, TfR1 is not recycled back to the cell surface leading to decreased cell surface TfR1 levels (95, 96) and lethal iron deprivation that can be rescued by the supplementation of iron (96). Our group has also developed a similar antibody-avidin fusion protein targeting the rat TfR1 (chOX26Av), also known as anti-rat TfR IgG3-Av (145). This fusion protein delivers compounds into rat cancer cell lines and like its human counterpart, also has direct cytotoxic activity against rat myeloma cells Y3-Ag1.2.3 and the rat T-cell lymphoma cell line C58 (NT) D.1.G.OVAR.1 (145). Importantly, this fusion protein was shown to exist as a dimer in solution due to the tetrameric form of avidin (145). Therefore, the enhanced *in vitro* cytotoxic effect of ch128.1Av compared to its parental antibody may be due, at least in part, to the increased valency leading to increased cross-linking of TfR1 on the surface of cancer cells.

**Figure 6:**
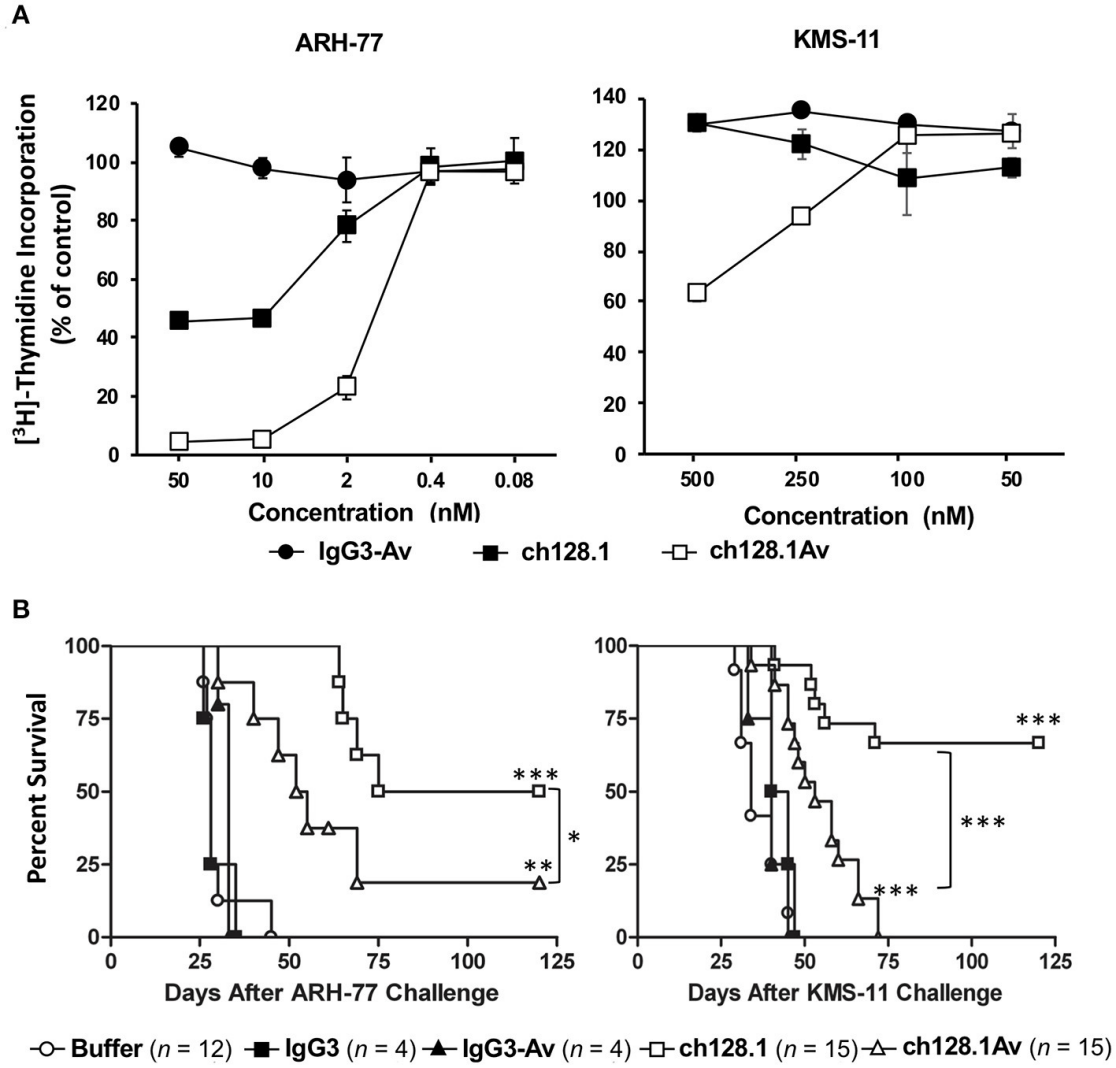
Anti-tumor activity of ch128.1 and ch128.1Av *in vitro* and in disseminated xenograft mouse models of MM. **(A)** Thymidine incorporation assay: Cells were treated with various concentrations (ranging from 0.08 to 50 nM for MM ARH-77 cells and 50 to 500 nM for MM KMS-11 cells) of ch128.1/IgG3, ch128.1Av, or an isotype control fusion protein (IgG3-Av) for a total of 96 h. Proliferation was measured by adding ^3^H-thymidine in the last 16–18 h. Data are presented as a percent of radioactivity incorporated into control cells. The average of triplicate wells is shown and error bars indicate the standard deviation. These results are representative of three independent experiments. **(B)** *In vivo* efficacy in two disseminated models of MM. Kaplan–Meier plots indicating survival of SCID-Beige mice challenged i.v. with 5 × 10^6^ ARH-77 (left panel) or KMS-11 (right panel) cells. For experiments with ARH-77 cells 100 μg of each treatment was injected i.v. 2 days after tumor challenge, whereas 125 μg of each treatment was used for the KMS-11 studies. Survival plots are the combined data of two experiments. *p < 0.05, **p < 0.01, and ***p < 0.001 (log-rank test) compared to buffer or the corresponding isotype control, except for the comparison between the antibody fusion protein and the parental antibody. Survival was based on the time from tumor challenge to the development of hind-limb paralysis (HLP), when mice were euthanized. This figure was produced by combining the data from Figures 3, 4 in Daniels et al. (144) with permission from Wolters Kluwer Health, Inc.

Despite the fact that ch128.1Av shows greater *in vitro* cytotoxic effect against cancer cells compared to its parental antibody, this was not observed in animal models. MM cells are dependent on an increased uptake of iron so treatments targeting iron homeostasis are good therapeutic options for this disease (54, 155). In order to evaluate the effects of ch128.1Av and its parental antibody *in vivo*, we used mice inoculated with either ARH-77 or KMS-11 cells since both are meaningful xenograft mouse models of MM (144, 156, 157). As shown in Figure 6A, ARH-77 cells are sensitive to the *in vitro* cytotoxicity of ch128.1Av and to a lesser extent to the effects of ch128.1 (144). KMS-11 cells are less sensitive to this effect and only ch128.1Av confers an effect at higher concentrations and thus, KMS-11 cells are considered to be non-sensitive cells. In disseminated xenograft models of human MM using either of these two cell lines inoculated i.v. in severe combined immunodeficiency mice with the beige (*Lyst*^*Bg*−*J*^) mutation (SCID-Beige), a single dose of either ch128.1 or ch128.1Av shows significant anti-tumor activity, although ch128.1 prolongs the survival of mice to a greater extent than the fusion protein (Figure 6B) (144). This was surprising given the fact that KMS-11 cells were not sensitive to the *in vitro* effects of either ch128.1Av or ch128.1. Anti-cancer activity of ch128.1 was also observed in a xenograft model of human AIDS-related non-Hodgkin lymphoma (AIDS-NHL) using the 2F7 human AIDS-related BL (AIDS-BL) cell line inoculated i.p. in NOD-SCID mice (142). Again, 2F7 cells are not sensitive to the *in vitro* cytotoxic effects of the antibody or the fusion protein (142). The increased *in vivo* anti-tumor activity of ch128.1 in these mouse models may be attributed to the lower bioavailability of ch128.1Av due to the presence of avidin that leads to its increased clearance from the blood and sequestration in the liver (158). The mechanism of the anti-tumor activity of ch128.1 in the xenograft MM model using the KMS-11 cell line was found to be dependent on the Fc region of the antibody since a mutant version (L234A/L235A/P329S) of the antibody with impaired ability to elicit effector functions did not confer protection (143). The anti-tumor activity of ch128.1 was also shown to be dependent, at least in part, on macrophages, but does not involve complement (143), although the antibody is capable of eliciting both ADCC and CDC *in vitro* (159). Whether this Fc-mediated activity is due to ADCC/ADCP and/or if there is a contribution of the inhibition of internalization of TfR1 into cancer cells when the antibody is simultaneously bound to FcγRs on immune cells in the tumor microenvironment remains to be determined. Subsequently, ch128.1 prolongs survival of mice in both early and late-stage disease in the xenograft mouse models bearing disseminated KMS-11 cells (143).

**Figure 7:**
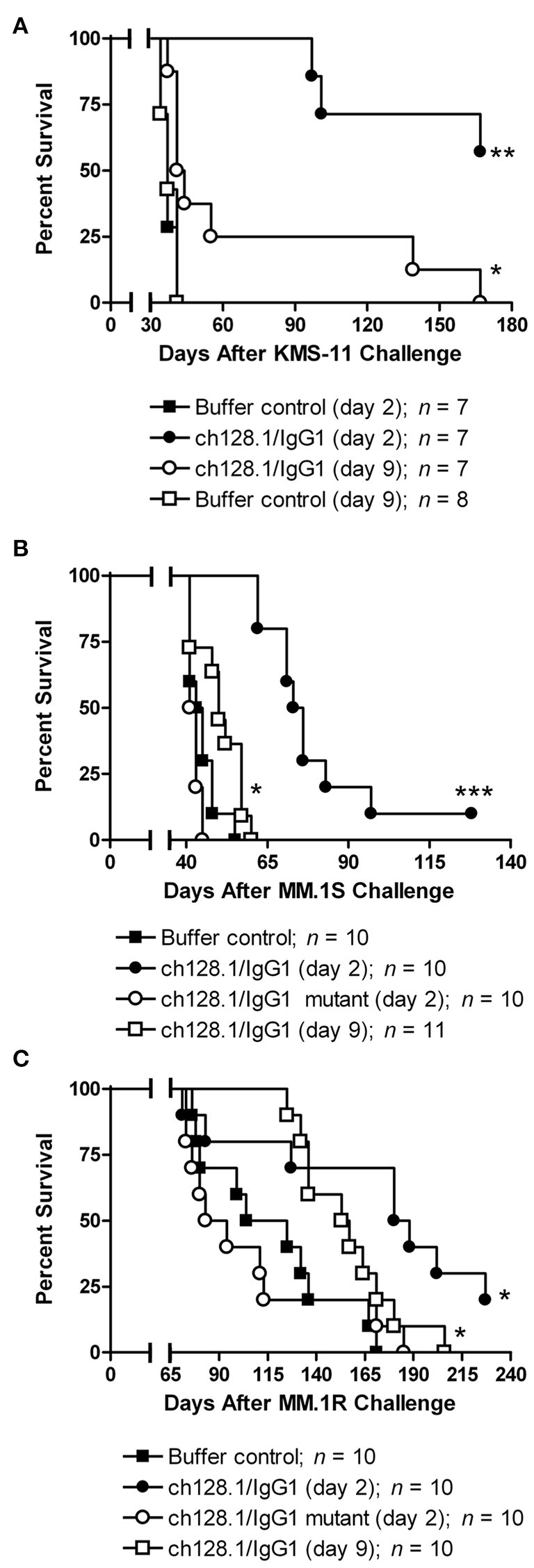
Anti-tumor activity of ch128.1/IgG1 in disseminated xenograft mouse models of MM. **(A)** SCID-Beige mice were challenged i.v. with 5 × 10^6^ KMS-11 cells and 2 or 9 days later treated i.v. with 100 μg ch128.1/IgG1. Mice were also challenged i.v. with 5 × 10^6^ **(B)** MM.1S or **(C)** MM.1R cells. cells. Two days after tumor challenge, mice were treated i.v. with 100 μg ch128.1/IgG1 or the ch128.1/IgG1 Fc mutant L234A/L235A/P329S. For each cell line (MM.1S and MM.1R), one group of mice was treated with 100 μg ch128.1/IgG1 9 days after tumor challenge. *p < 0.05, **p < 0.001, and ***p < 0.0001 (log-rank test). This figure was reproduced from Daniels-Wells et al. (163) with permission from Wolters Kluwer Health, Inc. The Creative Commons license does not apply to this content. Use of the material in any format is prohibited without written permission from the publisher, Wolters Kluwer Health, Inc. Please contact permissions@lww.com for further information.

The IgG1 isotype has been the choice for antibodies targeting malignant cells in the clinic that require an active Fc region to mediate antibody effector functions (97, 160). For this reason, combined with the potential liabilities of the IgG3 isotype, namely a shorter half-life in the blood, non-established manufacturability techniques, increased immunogenicity due to the increased number of allotypes compared to IgG1 (161), and proteolysis issues due to the extended hinge region (162), our group has developed an IgG1 version of the ch128.1 antibody (ch128.1/IgG1) (163). This IgG1 antibody also shows anti-cancer activity in the disseminated mouse model of MM using SCID-Beige mice and the human MM cell line KMS-11 (Figure 7A). This activity is similar to that of the IgG3 version and is observed in both the early and late-stage disease settings, although the anti-tumor activity is less in the late-stage model, as expected since the tumor burden is greater. Furthermore, ch128.1/IgG1 also prolonged the survival of SCID-Beige mice inoculated with human MM.1S (Figure 7B) or MM.1R (Figure 7C) cells. Both of these cell lines are of African American origin, which is of significance because the incidence and mortality rates of MM in individuals of African ancestry is higher compared to other races (164). The KMS-11 cell line is of Asian origin, for comparative purposes. The MM.1R cell line is a variant of MM.1S that is resistant to the glucocorticoid dexamethasone. The ability of ch128.1/IgG1 to prolong the survival of mice bearing MM.1R tumors is noteworthy since dexamethasone is a common treatment for MM and resistance to this therapeutic is well-known (165). Like the IgG3 version of this antibody, a mutant version of ch128.1/IgG1 (L234A/L235A/P329S) that has impaired effector functions failed to protect mice from MM.1S and MM.1R tumor development (Figures 7B,C) (163). This indicates that the Fc region of the antibody, similar to its ch128.1/IgG3 counterpart, is crucial for the anti-cancer effect of ch128.1/IgG1 in these MM mouse models. The anti-cancer effects of ch128.1/IgG1 in the MM.1S xenograft model of MM can be further enhanced using combination therapies consisting of either bortezomib or lenalidomide, common treatments for MM that are used in the clinic (166).

A humanized antibody (hu128.1) version of ch128.1/IgG1 was recently developed. Both ch128.1/IgG1 and hu128.1 show anti-cancer activity in xenograft models of human AIDS-NHL in SCID-Beige mice using both disseminated (cells injected i.v.) and local (cells injected s.c.) models of AIDS-NHL (167). For these studies two different human AIDS-NHL cell lines were used: 2F7-BR44, Epstein-Barr virus (EBV) positive BL cells that form metastases in the brain of mice upon i.v. inoculation, or JB cells, which are also relevant since they are BL cells that are EBV negative (167). In addition, ch128.1/IgG1 inhibits EBV-driven transformation of human B cells and lymphomagenesis *in vivo* using an immunodeficient NSG mouse model (168).

Taken together, these data show that targeting TfR1 with antibodies, such as ch128.1/IgG1 or hu128.1, is a promising therapeutic strategy for B-cell malignancies, including MM and NHL. Interestingly, the ch128.1/IgG1 antibody has also been incorporated into a nanobioconjugate and used to target human brain tumors and deliver an anti-cancer agent in mice (169). This different approach emphasizes the versatility of antibodies targeting TfR1.

#### Single Chain Fv (scFv) Fragments

To maximize the human content of antibodies, development of fully human monoclonal antibodies was possible with the advent of human scFv phage display libraries and transgenic mice bearing the human antibody repertoire (97, 98). Human full-length antibodies targeting TfR1 are discussed in the next section. Several human scFv fragments targeting TfR1 have been identified from phage libraries and evaluated as direct anti-cancer agents.

3TF12 and 3GH7 are neutralizing human scFv originally identified for their ability to bind and be rapidly internalized by human TfR1 (170). They were shown to have anti-proliferative activity, consistently inhibiting the *in vitro* proliferation and decreasing the viability of human hematopoietic cancer cell lines, such as Jurkat, ERY-1 and K562 erythroleukemic cells, HL-60 APL cells, and Raji BL cells, as well as U937, a histiocytic lymphoma cell line (170). This anti-proliferative effect in lymphoma and leukemia cells was improved by engineering bivalent versions of 3TF12 and 3GH7 scFv fragments, resulting in F12CH and H7CH respectively (170). F12CH and H7CH showed a stronger ability to inhibit Tf binding to the receptor and conferred more potent cytotoxic effects against ERY-1 cells than the original monovalent scFv. In Raji cells, treatment with these bivalent proteins induces signs of cell death via externalization of phosphatidylserine (PS) and increased granularity, although loss of mitochondrial membrane potential was not observed (170). In addition, large vacuoles were visible, which may indicate the induction of autophagy by the bivalent antibody fragments. The mechanism of the *in vitro* inhibition of proliferation involves inhibition of receptor function and iron deprivation, as iron supplementation reverses this cytotoxicity (170). In ERY-1 cells, the bivalent antibody fragments induced apoptosis as evident by externalization of PS, mitochondrial membrane depolarization, and cell size decrease. F12CH, which cross-reacts with mouse TfR1, was shown to reduce s.c. implanted tumor growth in a xenograft model (170). In this study, athymic *nu/nu* mice were challenged s.c. with ERY-1 cells. When tumors reached about 200 mm^3^ in volume, mice were treated twice a week with 200 μg of F12CH administered i.p. for a total of seven treatments over a 3-week period. Weight loss in treated mice was similar to that of control mice, suggesting that the antibody fragment was well-tolerated (170).

scFv-HAK is a human scFv “intrabody” that is specific for human TfR1 (171). Intrabody, or intracellular antibody, is a different therapeutic approach that employs the intracellular expression of an antibody or antibody fragment (such as scFv) and is directed to a specific location within the cell to elicit its effect by binding to its targeted antigen (172). scFv-HAK was designed to block TfR1 surface expression by localizing to the endoplasmic reticulum (ER) and binding to the receptor intracellularly, ultimately inducing iron deprivation and inhibition of proliferation. To that aim, it has been demonstrated to localize to the ER, reduce TfR1 surface expression, induce G1 cell cycle arrest, and induce apoptosis of MCF-7 human breast cancer cells *in vitro* (171).

#### Fully Human Anti-TfR1 Antibodies

scFv isolated from phage libraries can be retro-engineered to produce fully human antibodies that minimize immunogenicity. Fully human antibodies can also be produced using transgenic mice bearing the human antibody repertoire (97, 98). Three fully human anti-human TfR1 antibodies have been developed and their anti-cancer properties are discussed below. All were produced using the phage display technology.

Anti-TFRC is a neutralizing fully human IgG1 monoclonal antibody specific for human TfR1 that was generated using the phage display technology (41). This antibody showed anti-cancer effects against human oral squamous cell carcinoma (OSCC), inhibiting the *in vitro* proliferation in the OSCC cell lines HSC2, HSC3, SAS, and HSC4 as compared to the human keratinocyte cell line HaCaT (normal skin cells) and inducing apoptosis in HSC2 and SAS cells (41). The anti-TFRC antibody also showed the ability to induce ADCC against OCSS cells, which is influenced by TfR1 receptor expression levels, with OCSS cell lines showing high levels of TfR1 expression being the most affected by ADCC (41). Importantly, this fully human antibody also showed anti-cancer affects *in vivo* in a xenograft model of OCSS (41). This antibody, administered either 7.5 or 15 mg/kg i.v. two times per week for 3 weeks, inhibited the growth of s.c. inoculated SAS tumors in Rag-2/Jak3 double-deficient BALB/c mice.

**Figure 8:**
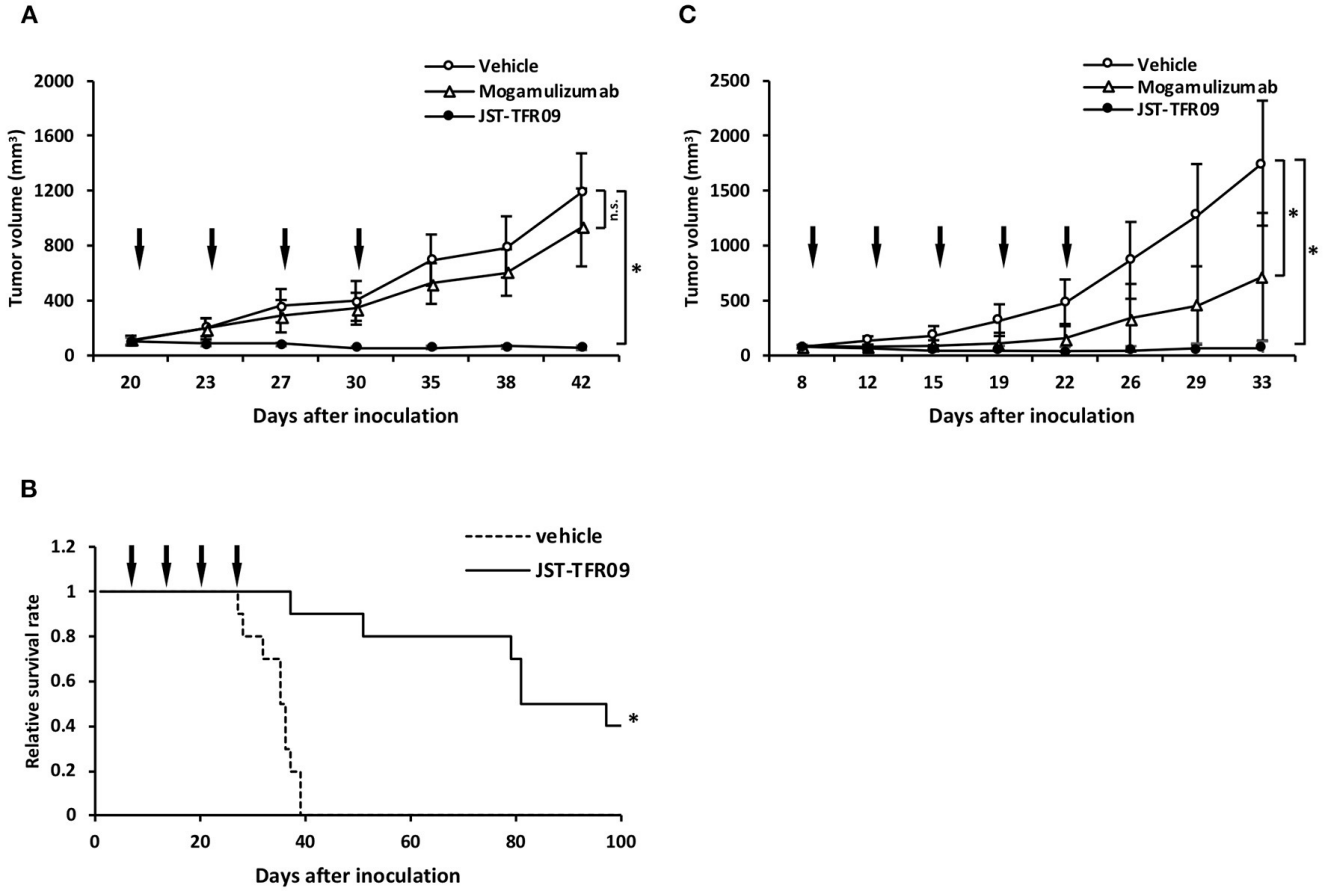
Anti-tumor activity of the fully human JST-TFR09 antibody in xenograft mouse models. **(A)** ATLL HTLV-1-infected MT-2 s.c. tumor volumes in NOG mice treated with 10 mg/kg JST-TFR09, mogamulizumab, or vehicle control (i.v.). Arrows indicate the day of injection. *p < 0.05 compared to the vehicle control group. **(B)** Kaplan–Meier survival curves of NOG mice bearing MT-2 cells, treated with JST-TFR09 or vehicle control. *p < 0.05 (log-rank test) compared to the vehicle control. **(C)** Cutaneous T-cell lymphoma HH s.c. tumor volume in SCID mice treated with 20 mg/kg of JST-TFR09, mogamulizumab, or vehicle. The arrow indicates the time of antibody injection. *p < 0.05 compared to the vehicle control. Mogamulizumab was added as an antibody with known ADCC activity. This figure was reprinted from Shimosaki et al. (173) with permission from Elsevier.

JST-TFR09 (also known as PPMX-T003) is another neutralizing fully human IgG1 monoclonal antibody specific for human TfR1 that was developed via phage display technology by the same group that developed the anti-TFRC antibody (173, 174). This antibody shows direct *in vitro* cytotoxic activity against a panel of six ATLL cell lines and primary cancer cells isolated from ATLL patients (173). JST-TFR09 showed cytotoxicity against human K562 erythroleukemic cells, HL-60 leukemic cells, and SU-DHL-2 human anaplastic large cell lymphoma cells. This cytotoxic effect was shown to be greater than that of the murine IgA antibody 42/6 (173). This antibody also induced ADCC against ATLL cell lines *in vitro* and demonstrated anti-cancer effects *in vivo* in several xenograft tumor models, including local and disseminated tumor models. In NOD/Shi-scid/IL-2Rγ^null^ (NOG) mice inoculated s.c. with the ATLL HTLV-1-infected cell line MT-2, i.v. treatment with 10 mg/kg JST-TFR09 4 times every 3 days starting at 20 days after tumor implantation blocked tumor growth (Figure 8A) and prolonged survival (Figure 8B) (173). In a similar model, this antibody was administered at 20 mg/kg i.v., 5 times every 3 days starting at 8 days after tumor implantation also blocked tumor growth in SCID mice bearing s.c. human cutaneous T-cell lymphoma HH tumors (Figure 8C) (173, 174). In a disseminated ATLL model using the MT-2 cell line injected i.v. into NOG mice, mice treated i.v. with 10 mg/kg JST-TFR09 2 times per week for 2 weeks starting at day 3 lived significantly longer than mice inoculated with vehicle control (Figure 8C). This increased survival was replicated in another experiment using the ATLL cell line Su9T01 under similar conditions (173, 174). This antibody was subsequently shown to have anti-cancer activity *in vivo* in SCID mice bearing s.c. tumors of human SU-DHL-2 (large cell lymphoma cells), Kasumi-1 or HL-60 (AML cells), or K562/ADM (erythroleukemic cells that are resistant to adriamycin treatment) (174). Moreover, in a disseminated model of ALL using SCID mice inoculated with the human CCRF-CEM, JST-TFR09 administered twice weekly for 3 weeks starting 3 days after tumor inoculation significantly prolonged animal survival (174). Furthermore, JST-TFR09 showed anti-cancer activity in an AML patient-derived xenograft (PDX) model. For this study NOG mice were implanted with AML cells intratibially. The next day mice were treated with 10 mg/kg JST-TFR09 i.v. once weekly for 4 weeks (174).

The third fully human antibody that has been developed is the H7 neutralizing IgG1 antibody (H7-IgG1) for which the variable regions from the 3GH7 scFv described above were used to produce a human IgG1 antibody (175). Another version of this antibody was produced containing two scFvs linked to the Fc region of human IgG1 (H7-Fc). Interestingly, only the H7-Fc was able to bind human and mouse TfR1, while the full length H7-IgG1 antibody was only able to bind human TfR1. Both forms of the H7 antibody inhibited the *in vitro* growth and induced apoptosis in human ERY-1 erythroleukemia cells, and human Raji BL cells (175). Both formats also induced ADCC activity, but the H7-IgG1 showed more potent ADCC activity (175). Tumor regression was observed in nude mice treated with 100 μg H7-Fc (i.p injection twice a week for 4 weeks) bearing ERY-1 erythroleukemia cells xenografts, but significant prolongation of survival was not observed (175). No toxicity was observed in the mice treated with H7-Fc. The authors also noted that the H7-Fc, through its interaction with the TfR1, was protected from elimination and seemed to persist *in vivo* through an FcRn-like mechanism (175). The activity of the H7-IgG1 (200 μg by i.p injection twice a week for 4 weeks) was stronger in this mouse model with the IgG1 prolonging animal survival. The stronger activity of the IgG1 vs. H7-Fc was attributed by the authors to its ability to induce ADCC at higher levels compared to H7-Fc. However, the authors also note that the decreased activity of H7-Fc *in vivo* could be linked to the decreased accumulation of the H7-Fc in the tumor due to the fact that it cross-reacts with mouse TfR1 and potentially localized to other tissues. The mechanism of *in vivo* anti-cancer activity was considered to be both through iron deprivation and ADCC (175).

## Toxicity Concerns

Normal cells that express high levels of TfR1 may also be targeted by anti-TfR1 antibodies leading to unwanted toxicities. Cells that are of particular concern include hematopoietic committed progenitor cells expressing high levels of TfR1 such as erythroblasts, that require iron for heme synthesis (11, 12, 20, 176). For most of the studies mentioned above using antibodies or antibody fragments targeting human TfR1, the xenograft mouse models used to determine anti-tumor efficacy are not useful for evaluating toxicity to normal cells due to the lack of cross-reactivity with mouse TfR1. Despite this fact, these models have been used in the preclinical development of antibodies currently being used in the clinic and they can still be relevant in the preclinical development of antibodies that do not cross-react with their murine antigen counterpart (177–179). In the studies described above only the neutralizing F12CH bivalent scFv and the neutralizing H7-Fc antibodies cross-react with mouse TfR1 and thus, toxicity to normal tissues could be assessed in the xenograft mouse models. Weight loss was not observed in mice treated with either construct suggesting that both were well-tolerated in these mouse models (170, 175). However, only a single dosing scheme of these antibodies was tested and toxicity evaluation was restricted to monitoring weight loss. Additionally, since H7-Fc antibody did not display the same level of ADCC and anti-tumor activity compared to the full length H7-IgG1 antibody the value of this study on the toxicity of targeting TfR1 is limited. Other techniques and animal models discussed below have been used to evaluate the potential toxicity of anti-TfR1 antibodies to normal cells.

Toxicity to red blood cell progenitors has been documented with the use of a non-neutralizing mouse/human chimeric IgG1 antibody targeting murine TfR1 (anti-TfR^D^) (180). C57BL/6 mice given more than 1 mg/kg of the antibody i.v. showed a reduction in the number of reticulocytes, profound lethargy, and occasional limb and whole body spastic movements in a few animals within 5 min of dosing (180). After about 20 min, the mice were no longer lethargic, but developed a hunched appearance with some mice voiding reddish brown urine, consistent with hemolysis and hemoglobinuria. However, this effect was completely reversible and mice appeared normal 1–2 h after dosing. Additionally, a decrease in circulating reticulocytes and those in the bone marrow was also observed within 1 h of dosing. These effects were attributed to the effector functions (ADCC and/or CDC) of the antibody (180). *In vitro* toxicity to erythroblasts as also been reported with the fully human IgG1 neutralizing antibody JST-TFR09 targeting human TfR1 (173).

Toxicities to myeloid and erythroid progenitor cells have also been documented using other anti-TfR1 antibodies. The murine antibodies 42/6 (neutralizing), B3/25 (non-neutralizing), and 43/31 (neutralizing) targeting human TfR1 were shown to induce reversible, dose-dependent inhibition of CFU-GM *in vitro* (126). The IgA antibody 42/6 was the most potent of the three in inducing these *in vitro* effects. The non-neutralizing RI7 208 rat IgM anti-mouse TfR1 antibody administered i.p. into AKR/J mice (3 mg twice weekly up to eight total doses) did not show evidence of gross toxicity, decreased red or white blood cell count, nor evidence of cellular damage in tissue sections of the small intestine, liver, or spleen (116). In mice given 1 mg of rat IgM antibody RI7 208 i.p. daily for seven days, a two-fold decrease in the number of erythroid progenitor colony formation units (CFU-e) in the bone marrow was observed (116). The non-neutralizing RI7 217 IgG2a rat anti-mouse TfR1 antibody administered i.p. was reported to be non-toxic to CD-1 mice at a single dose of 25–50 mg/kg, while the same antibody conjugated to an immunotoxin was toxic (181).

The effect of anti-TfR1 antibodies on lymphoid cells has also been evaluated. The rat anti-mouse TfR1 IgG2a antibody C2F2 is a non-neutralizing antibody that preferentially inhibited IL-1-dependent T-cell activation but did not show significant effects on interleukin-2 (IL-2) dependent T-cell activation *in vitro* (182). This antibody also inhibited PHA-stimulated growth of murine C3H/HeJ T cells, concanavalin A stimulation to some extent, but not lipopolysaccharide stimulation of lymphocyte activation *in vitro* (182). The mouse/human chimeric IgG1 antibody D2C, that targets the human receptor, inhibited the proliferation of PHA-activated peripheral blood mononuclear cells (PBMCs) *in vitro* in a dose-dependent manner, while no effect was observed on unstimulated PBMCs (183). A similar *in vitro* cytotoxic effect on activated lymphocytes, but not resting cells, has been reported with other anti-TfR1 antibodies including the murine IgM anti-human TfR1 antibody RBC4, the murine IgG2b anti-human TfR1 neutralizing antibody A24, and the fully human IgG1 JST-TfR09 neutralizing antibody (94, 135, 173).

It is important to note that progenitor cells above are committed progenitor cells and are known to express high levels of TfR1 (11, 12, 20, 176). However, pluripotent (non-committed) hematopoietic stem cells (HSC) express low to undetectable levels of TfR1 (176, 184, 185). Interestingly, treatment of HSC with 10 nM of the mouse/human chimeric IgG3 ch128.1Av fusion protein alone (or even complexed with the potent plant toxin saporin) showed no toxicity to this population of cells due to the lack of expression of TfR1 (149). In contrast, 2.5 nM ch128.1Av was toxic to committed progenitor cells such as CFU-e, erythroid burst formation units (BFU-e), and CFU-GM (153). Taken together all of the above studies suggest that even though committed progenitor cells are vulnerable to anti-TfR1 treatment, HSC are not and thus, can repopulate the populations that are lost.

Toxicity of anti-TfR1 antibodies in cynomolgus monkeys (*Macaca fasicularis*) has been reported for two anti-TfR1 antibodies. In the first study the murine IgG2b A24 antibody targeting human TfR1 caused a slight decrease of hemoglobin levels and decreased serum iron levels in these monkeys. Additionally, apoptosis was observed in the germinal center of lymph nodes where the B-cell and T-cell rate of proliferation is high (186). In the second study, cynomolgus monkeys given multiple doses of 30 mg/kg of the fully human IgG1 JST-TFR09 antibody (also known as PPMX-T003) showed moderate anemia (173, 174). However, no other toxicities were observed in the animals of both studies. In the Phase I clinical trial of the murine IgA 42/6 antibody, treatment was generally well-tolerated (131). One patient that received two treatments of the antibody developed an “allergic-type response” that was attributed to the HAMA response. Additionally, an initial decrease in BFU-e growth was observed; however, this decrease was not significant.

Taken together, these studies suggest that anti-TfR1 antibodies may have an effect on certain normal cells, especially immature reticulocytes and other hematopoietic progenitor cells as well as immune cells that are activated and express high levels of TfR1. These studies also suggest that several factors may contribute to potential toxicity including the class and species of the antibody, the targeted epitope, binding affinity, valency, effector functions, route of administration, dose and schedule of treatment, as well as the animal model used to assess toxicity. Thus, it is important that the safety of each antibody be carefully evaluated individually in each preclinical and clinical setting.

## Concluding Remarks

TfR1 is a meaningful anti-cancer target due to its overexpression on malignant cells and its central role in cancer cell pathology. Antibodies against TfR1 have been used for years to deliver therapeutic agents into cancer cells. In addition, the concept of using antibodies targeting TfR1 as direct anti-cancer agents is not new, however, it has been reignited and continues to gain interest given the recent encouraging data with several of these antibodies. Advances in genetic engineering are making safer mediators of this treatment strategy, with increasingly more “human” antibodies with potentially less side effects and increased potency. These anti-TfR1 antibodies with human constant regions have potent Fc-mediated effector functions against cancer cells and are less susceptible to the common drawbacks of using rodent antibodies in humans. Anti-tumor activity has been demonstrated in a wide range of cancer types, both hematological as well as solid cancers even though hematopoietic malignancies have shown particular susceptibility. Various anti-TfR1 antibodies exhibit significant anti-cancer activity through different mechanisms and provide a manageable toxicity profile. The development of antibodies targeting the TfR1 as direct cancer therapeutics is a continuously growing field, paving the way to clinical trials.

## Author Contributions

MLP and TD-W devised the concept and structure of the review article and contributed to the overall direction and structure of the manuscript. PC, LL, and TD-W conducted the literature searches, wrote the initial draft of the manuscript, and prepared the figures. All authors edited the manuscript and agreed on the final content.

## Conflict of Interest

MLP has financial interest in Stellar Biosciences, Inc. The Regents of the University of California are in discussions with Stellar Biosciences to license a technology invented by MLP to this firm. In addition, MLP has a financial interest in Klyss Biotech, Inc. The remaining authors declare that the research was conducted in the absence of any commercial or financial relationships that could be construed as a potential conflict of interest.
