# Supplementary material for: Antibodies Targeting the Transferrin Receptor 1 (TfR1) as Direct Anti-cancer Agents
Source: Front Immunol. 2021 Mar 17;12:607692. doi: 10.3389/fimmu.2021.607692 (PMC8010148; doi:10.3389/fimmu.2021.607692)
Supplement: Supplementary file 1 [file Table_1.pdf]

Supplementary Table I: Anti-TfR1 antibodies that show direct anti-cancer activity.

Antibody name	Class and subclass	Targeted TfR1 species	Inhibits binding of Tf *	Anti-cancer Activity	References
RI7 208	Rat IgM	Mouse	No	***In vitro***<br>Inhibits proliferation of murine myeloma and lymphoma cell lines.<br>***In vivo***<br>Increases survival in a syngeneic murine model of leukemia (s.c. inoculation).	(92, 114-116)
REM 17.2	Rat IgM	Mouse	No	***In vitro***<br>Inhibits cancer cell proliferation.	(92,116)
RI7 217	Rat IgG2a	Mouse	No	***In vitro***<br>Inhibits proliferation of a murine T-cell lymphoma cell line, enhanced in the presence of a cross-linking antibody.	(92)
D18	Mouse IgG2a	Chicken	No	***In vitro***<br>Inhibits proliferation and induces caspase-independent cell death in a chicken B-lymphoid tumor cell line.	(117-118)
D19	Mouse IgG2a	Chicken	No	***In vitro***<br>Inhibits proliferation and induces caspase-independent cell death in a chicken B-lymphoid tumor cell line.	(117-118)
7579	Mouse IgG	Human	ND	***In vitro***<br>Inhibits proliferation and induces apoptosis of glioma, hepatoma, and breast cancer cell lines.<br>In combination with curcumin induces necrosis in glioma cell lines.<br>In combination with nimustine induces synergistic cytotoxic effects in glioma cell lines.<br>In combination with 5-fluorouracil, synergistically increases necrosis of breast and hepatocellular cancer cell lines.<br>In combination with doxurubicin, additively increases necrosis of breast and hepatocellular cancer cell lines.<br>In combination with sinomenine hydrochloride, decreases proliferation and induces apoptosis in a hepatocellular cancer cell line.	(120-122, 132)
E2.3	Mouse IgG1	Human	ND	***In vitro***<br>Inhibits proliferation of MM and breast cancer cell lines in combination with the antibody A27.15.	(125, 133)
A27.15	Mouse IgG1	Human	No	***In vitro***<br>Inhibits proliferation of MM and breast cancer cell lines in combination with the antibody E2.3.<br>***In vivo***<br>In combination with the antibody D65.30 inhibits T-cell leukemia tumor growth in a murine xenograft model.	(125, 133)

B3/25	Mouse IgG1	Human	No	***In vitro***<br>Inhibits proliferation of human AML cell lines.	(126)
43/31	Mouse IgG	Human	Yes	***In vitro***<br>Inhibits proliferation of human AML cell lines.	(126)
D65.30	Mouse IgG1	Human	No	***In vitro***<br>Inhibits proliferation of a human ALL (T-cell origin) cell line.<br>***In vivo***<br>Limited inhibition of s.c. implanted human ALL (T-cell origin) tumor growth in a murine xenograft model as a single agent and with increased effectiveness when combined with the antibody A27.15.	(127)
A24	Mouse IgG2b	Human	Yes	***In vitro***<br>Inhibits proliferation and induces apoptosis of ATLL, AML, and MCL cell lines.<br>***In vivo***<br>Abrogates human AML and MCL tumor formation in a s.c. mouse xenograft model. Inhibits tumor growth in a delayed tumor growth model.	(94, 119, 128)
RBC4	Mouse IgM	Human	ND	***In vitro***<br>Inhibits proliferation and induces cell death in human CML, ALL (T-cell origin), TCL, and AML cell lines.	(135)
42/6	Mouse IgA	Human	Yes	***In vitro***<br>Inhibits proliferation of human ALL (T-cell origin), AML, and melanoma cell lines.<br>***In vivo***<br>Phase I clinical trial showed out of 27 patients with various malignancies, 3 showed mixed anti-tumor response, one with follicular small cleaved cell lymphoma, one with HL, and one with CLL.	(124, 126-127,130,-131, 136)
D2C	Mouse/human chimeric IgG1 (Fv from 7579)	Human	ND	***In vitro***<br>Inhibits proliferation and induces apoptosis in an erythroleukemia cell line.<br>Induces ADCC against erythroleukemia, ALL (T-cell origin), and hepatocellular cancer cell lines.<br>Induces CDC against ALL (T-cell origin) and hepatocellular cancer cell lines.<br>In combination with curcumin, enhances anti-cancer activity in a castration resistant prostate cancer cell line.	(139-141)
ch128.1Av	Mouse/human chimeric IgG3-Avidin (Fv from 128.1)	Human	No	***In vitro***<br>Exhibits anti-proliferative/pro-apoptotic activity in human MM, AML, BL, B-lymphoblastoid cells, TCL, and erythroleukemia cell lines.<br>Enhances anti-cancer cytotoxic effects when used in combination with gambogic acid, HXR9, or cisplatin.<br>***In vivo***<br>Increases survival in a disseminated xenograft murine models of human MM, though less effective than ch128.1.	(96, 144, 152-154)
chOX26Av	Mouse/human chimeric IgG3-Avidin	Rat	No	***In vitro***<br>Exhibits anti-proliferative/pro-apoptotic activity in rat myeloma and T-cell lymphoma cell lines.	(145)

	(Fv from OX26)				
ch128.1/IgG3	Mouse/human IgG3 (Fv from 128.1)	Human	No	***In vitro***<br>Inhibits proliferation and induces apoptosis of certain hematopoietic malignant cell lines such as B-lymphoblastoid cell lines.<br>Induces ADCC and ADCP against malignant B cell lines.<br>***In vivo***<br>Increases survival in disseminated xenograft models of human MM. Increases survival in a xenograft model of AIDS-NHL where cells were inoculated i.p.	(95-96, 142, 144, 160)
ch128.1/IgG1	Mouse/human IgG1 (Fv from 128.1)	Human	No	***In vitro***<br>Induces ADCC and ADCP against a MM cell line.<br>***In vivo***<br>Increases survival in disseminated xenograft models of human MM and AIDS-NHL.<br>In combination with either lenalidomide or bortezomib, improves survival in disseminated xenograft models of human MM compared to the single agents alone.<br>Inhibits EBV-driven lymphomagenesis in mice bearing human B cells.	(163, 166-168)
hu128.1	Humanized IgG1 (Fv from 128.1)	Human	No	***In vivo***<br>Increases survival in disseminated xenograft models of human AIDS-NHL.	(167)
3TF12	Fv	Human	Yes	***In vitro***<br>Inhibits proliferation and decreased viability of human TCL, erythroleukemia, BL, histiocytic lymphoma, and AML cell lines.	(170)
3GH7	scFv	Human	Yes	***In vitro***<br>Inhibits proliferation and decreased viability of human TCL, erythroleukemia, BL, histiocytic lymphoma, and AML cell lines.	
H7CH	Bivalent scFv	Human	Yes	***In vitro***<br>Inhibits proliferation and induces apoptosis of an erythroleukemia cell line.	(170)
F12CH **	Bivalent scFv	Human	Yes	***In vitro***<br>Inhibits proliferation and induces apoptosis of an erythroleukemia cell line.<br>Inhibition of proliferation is rescued by iron supplementation.<br>***In vivo***<br>Inhibits tumor growth of erythroleukemia cells in a s.c. xenograft mouse model.	(170)
scFv HAK	Intra-cellular scFv	Human	Yes	***In vitro***<br>Inhibits proliferation and induces apoptosis in a human breast cancer cell line.	(171)
H7-Fc **	scFv-Fc ***	Human	Yes	***In vitro***<br>Induces apoptosis in human erythroleukemia and BL cell lines.<br>Induces ADCC against mastocytoma leukemic cells and lymphoma cell lines.<br>***In vivo***<br>Tumor regression was observed in a s.c. erythroleukemia xenograft mouse model.	(175)

Anti-TFRC	Fully human IgG1	Human	Yes	***In vitro***<br>Inhibits proliferation and induces apoptosis in human OSCC cell lines.<br>Induces ADCC against OSCC cell lines.<br>***In vivo***<br>Inhibits human OSCC tumor growth in a murine xenograft model.	(41)
JST-TFR09 (PPMX-T003)	Fully human IgG1	Human	Yes	***In vitro***<br>Inhibits proliferation and increases apoptosis in ATLL, AML, erythroleukemia, and anaplastic large cell lymphoma cell lines.<br>Induces ADCC against an ATLL cell line.<br>***In vivo***<br>Inhibits human ATLL, cutaneous T-cell lymphoma, large cell lymphoma, AML, and erythroleukemia tumor growth in s.c. xenograft mouse models.<br>Increases survival in disseminated xenograft mouse models of ATLL and ALL (T-cell origin).<br>Showed anti-cancer activity in xenograft mouse models using patient-derived AML cells implanted intratibially.	(173-174)
H7-IgG1	IgG1 (Fv from 3GH7 scFv)	Human	Yes	***In vitro***<br>Induces apoptosis in human erythroleukemia and BL cell lines.<br>Induces ADCC of mastocytoma leukemic cells and lymphoma cell lines.<br>***In vivo***<br>Tumor regression and increased survival in a s.c. erythroleukemia xenograft mouse model.	(175)

** If the antibody inhibits the binding of Tf it is known as a neutralizing antibody.*
*** These antibodies cross-react with mouse TfR1.*
**** Two 3GH7 scFv attached to the Fc region of human IgG1.*
ADCC, antibody-dependent cell-mediated cytotoxicity; ADCP, antibody-dependent cell-mediated phagocytosis; AIDS-NHL, acquired immunodeficiency syndrome associated non-Hodgkin lymphoma; ALL, acute lymphocytic leukemia; AML, acute myeloid leukemia; ATLL, adult T-cell leukemia/lymphoma; BL, Burkitt lymphoma; CDC, complement-mediated cytotoxicity; CLL, chronic lymphocytic leukemia; CML, chronic myeloid leukemia; Fv, variable regions; HL, Hodgkin lymphoma; i.p., intraperitoneal; MCL, mantle cell lymphoma; MM, multiple myeloma; ND, not described; OSCC, oral squamous cell carcinoma; s.c., subcutaneous, TCL, acute T-cell leukemia.
